# RNA-binding protein ZCCHC4 promotes human cancer chemoresistance by disrupting DNA-damage-induced apoptosis

**DOI:** 10.1038/s41392-022-01033-8

**Published:** 2022-07-20

**Authors:** Ha Zhu, Kun Chen, Yali Chen, Juan Liu, Xiaomin Zhang, Yumei Zhou, Qiuyan Liu, Bingjing Wang, Taoyong Chen, Xuetao Cao

**Affiliations:** 1 National Key Laboratory of Medical Immunology, Institute of Immunology, Second Military Medical University, 200433 Shanghai, China; 2 Department of Immunology, Center for Immunotherapy, Institute of Basic Medical Sciences, Peking Union Medical College, Chinese Academy of Medical Sciences, 100005 Beijing, China; 3 Frontier Research Center for Cell Response, Institute of Immunology, College of Life Sciences, Nankai University, 310071 Tianjin, China

**Keywords:** Cancer therapy, Molecular medicine

## Abstract

RNA-binding proteins (RBPs) play important roles in cancer development and treatment. However, the tumor-promoting RBPs and their partners, which may potentially serve as the cancer therapeutic targets, need to be further identified. Here, we report that zinc finger CCHC domain-containing protein 4 (ZCCHC4) is of aberrantly high expression in multiple human cancer tissues and is associated with poor prognosis and chemoresistance in patients of hepatocellular carcinoma (HCC), pancreatic cancer and colon cancer. ZCCHC4 promotes chemoresistance of HCC cells to DNA-damage agent (DDA) both in vitro and in vivo. HCC cell deficiency of ZCCHC4 reduces tumor growth in vivo and intratumoral interference of *ZCCHC4* expression obviously enhances the DDA-induced antitumor effect. Mechanistically, ZCCHC4 inhibits DNA-damage-induced apoptosis in HCC cells by interacting with a new long noncoding RNA (lncRNA) AL133467.2 to hamper its pro-apoptotic function. Also, ZCCHC4 blocks the interaction between AL133467.2 and γH2AX upon DDA treatment to inhibit apoptotic signaling and promote chemoresistance to DDAs. Knockout of ZCCHC4 promotes AL133467.2 and γH2AX interaction for enhancing chemosensitivity in HCC cells. Together, our study identifies ZCCHC4 as a new predictor of cancer poor prognosis and a potential target for improving chemotherapy effects, providing mechanistic insights to the roles of RBPs and their partners in cancer progression and chemoresistance.

## Introduction

RNA-binding proteins (RBPs) are essential in the maintenance of cell homeostasis by recognizing hundreds of transcripts and forming extensive regulatory networks with partner proteins and RNAs.^1^ Recently, the roles of RBPs and their partners in cancer progression and treatment including chemotherapy and chemoresistance attracted much attention.^2,3^ RBPs were once considered largely “undruggable”, but currently small molecules or chemically modified antisense oligonucleotides targeting RBPs have been characterized for the treatment of certain diseases.^4,5^ For example, recent studies have proposed several RBP-based treatments in breast cancer, such as spliceosome- and estrogen receptor α-targeted therapies.^6–8^ Besides, RBP ZFP36L2 has been reported as a myeloid leukemia differentiation regulator and a new therapeutic target.^9,10^ Therefore, identifying new RBPs and their partners which are associated with cancer progression and uncovering the underlying mechanisms may help to provide more potential therapeutic targets for the treatment of cancer patients.

Resistance to chemotherapy has long been the main obstacle of clinical cancer treatment.^11^ The intrinsic or acquired drug resistance limits the efficacy of chemotherapy in several solid cancers, including intractable hepatocellular carcinoma (HCC), which is one of the leading causes of cancer-associated mortality worldwide.^12,13^ Many biological or pathological mechanisms have been implicated in chemoresistance, including overexpression of anti-apoptotic proteins and aberrations in DNA-damage response (DDR).^14,15^ In addition, increasing evidence suggest epigenetic regulation may play a critical role in cancer cell chemoresistance.^16,17^ However, the epigenetic roles of RBPs and their partners in the chemoresistance of solid cancers remain poorly understood and thus worth further investigations.

We previously conducted a non-biased high-throughput RNA interfering screening of 711 epigenetic modifiers for their potential roles in interferon α (IFNα)-mediated inhibition of hepatitis B virus (HBV) replication in HCC cells, and discovered that 15 candidates might regulate IFNα antiviral activity.^18^ Beyond the function in antiviral infection, type I interferon (IFN-I) can also limit proliferation and drive senescence and apoptosis of cancer cells.^19^ Based on the previous screening results, we therefore went further to detect the antitumor effects of those candidates. Among 15 epigenetic regulators, we found that high expression of zinc finger (ZnF) CCHC domain-containing protein 4 (*ZCCHC4*) was correlated with poor prognosis of HCC patients, and *ZCCHC4* was significantly upregulated in HCC cells after treatment of DNA-damage agents (DDAs) including oxaliplatin (OXA), a preferred chemotherapeutic agent for patients with advanced HCC.^20^ The results indicate that ZCCHC4 might be involved in modulating the chemoresistance of HCC cells to DDA. As a canonical RBP containing consensus ZnF domain and m^6^A methyltransferase (MTase) domain, ZCCHC4 primarily mediates m^6^A methylation of 28S rRNA.^21–23^ Recent studies reported ZCCHC4 could facilitate tumorigenesis through yet to be identified molecular mechanisms.^21,24^ In our current research, we demonstrate that ZCCHC4 suppress DNA-damage-induced apoptosis in HCC cells via interacting with a new long noncoding RNA (lncRNA) AL133467.2 as well as DNA-damage indicator γH2AX in the nucleus. Our findings identify ZCCHC4 as a potential target to improve the chemotherapy effects, and provide insight into the role of RBP-RNA network in cancer progression.

## Results

### ZCCHC4 is highly expressed in human cancers and correlated with poor prognosis

**Fig. 1:**
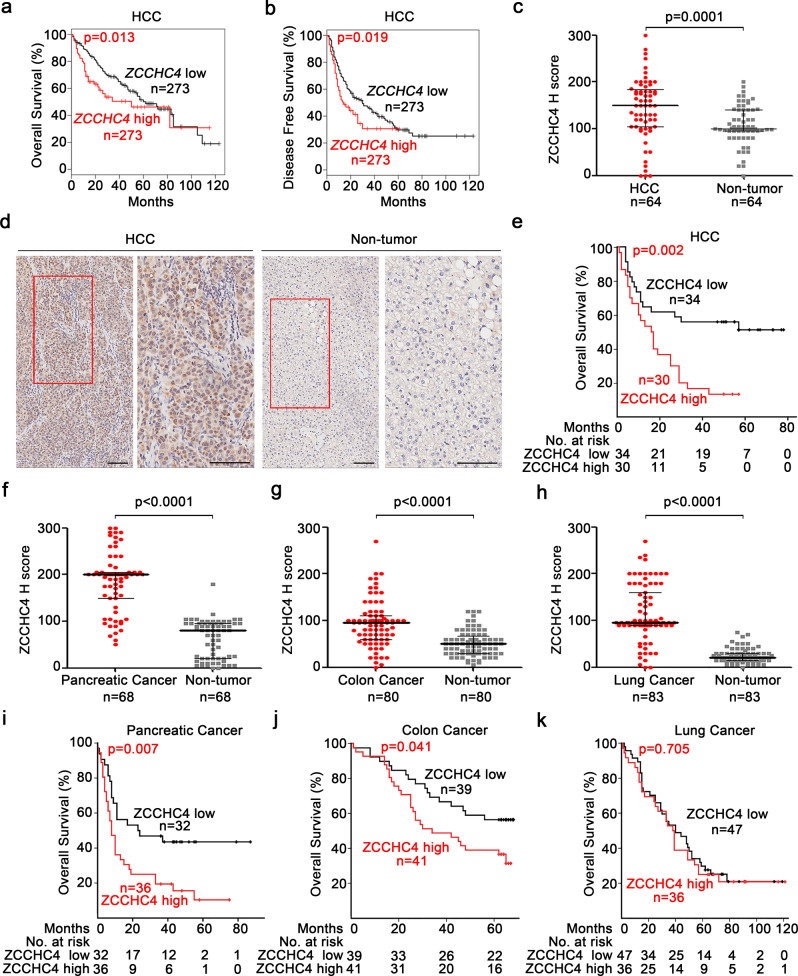
High expression of ZCCHC4 in human cancers predicts poor prognosis. **a**, **b** Overall survival (**a**) and disease-free survival (**b**) of HCC patients with high (*n* = 273) and low (*n* = 273) *ZCCHC4* mRNA expression (third quartile group cutoff) analyzed in GEPIA. **c** IHC examinations of ZCCHC4 protein expression in HCC (*n* = 64) and paired non-tumor (*n* = 64) samples. Median H score and interquartile range are shown and analyzed with paired Student’s *t* test. **d** Representative IHC images (scale bar = 100 μm) of ZCCHC4 protein in HCC and paired non-tumor biopsies (with red box region magnified; right). **e** Overall survival curve of HCC patients with high (*n* = 30) and low (*n* = 34) ZCCHC4 protein expression (median group cutoff). **f**–**h** ZCCHC4 protein expression examined by IHC in tumor and paired non-tumor samples (pancreatic cancer (*n* = 68) (**f**), colon cancer (*n* = 80) (**g**), and lung cancer (*n* = 83) (**h**). Median H score and interquartile range are shown and analyzed with paired Student’s *t* test. **i**–**k** Overall survival curve of pancreatic cancer patients (*n* = 68) (**i**), colon cancer patients (*n* = 80) (**j**) or lung cancer patients (*n* = 83) (**k**) with high and low ZCCHC4 protein expression (median group cutoff). The survival curves (**a**, **b**, **e**, and **i**–**k**) were analyzed with a Log-rank test

ZCCHC4 belongs to the zinc finger CCHC-type (ZCCHC) superfamily and is a newly discovered rRNA m^6^A MTase mainly localized in the nucleus.^21^ Here, we used Gene Expression Profiling Interactive Analysis (GEPIA) database^25^ and tissue microarray (TMA) to evaluate the clinical relevance of ZCCHC4. We found that the mRNA expression of *ZCCHC4* was higher in HCC tissues as compared to non-tumor tissues (Supplementary Fig. S1a), and the higher expression of *ZCCHC4* in HCC tissues predicted shorter overall survival and disease-free survival (Fig. 1a, b). Consistently, the immunohistochemistry (IHC) analysis showed that the protein level of ZCCHC4 was significantly higher in HCC tissues, with 73% (47/64) of HCC tissues showing increased ZCCHC4 expression compared to corresponding non-tumor tissues (Fig. 1c, d and Supplementary Fig. S1b, c). Correlation analysis indicated that HCC patients with higher protein expression of ZCCHC4 displayed shorter overall survival (Fig. 1e). Therefore, ZCCHC4 is aberrantly overexpressed in HCC tissues and is closely correlated with the poor prognosis of HCC patients.

In addition to HCC, IHC analysis of TMA showed significantly higher expression of ZCCHC4 in pancreatic cancer (PC), colon cancer (CC), and lung cancer (LC) tissues compared to that in their paired non-tumor tissues (Fig. 1f–h and Supplementary Fig. S1d–i). Notably, PC patients and CC patients with higher ZCCHC4 expression demonstrated shorter overall survival (Fig. 1i, j), while LC patients with higher ZCCHC4 expression did not show survival disadvantage (Fig. 1k). Thus, ZCCHC4 may play an important role in multiple cancer types.

### Deficiency of *ZCCHC4* reduces tumor growth of HCC in vivo

To investigate the role of ZCCHC4 in HCC, we generated three types of HepG2 cell lines with *ZCCHC4* knockout by CRISPR/Cas9 technology (ZCCHC4 KO1, KO2, and KO3) (Supplementary Fig. S2a, b). Then, we subcutaneously injected ZCCHC4 KO cells and wild-type HepG2 cells (WT cells) into nude mice and found that mice bearing ZCCHC4 KO cells displayed slower tumor growth and longer overall survival (Supplementary Fig. S2c–f). Thus, ZCCHC4 promotes tumor growth of HCC in vivo, indicating that ZCCHC4 is pro-tumorigenic in HCC.

### ZCCHC4 promotes chemoresistance of HCC cells in vitro

**Fig. 2:**
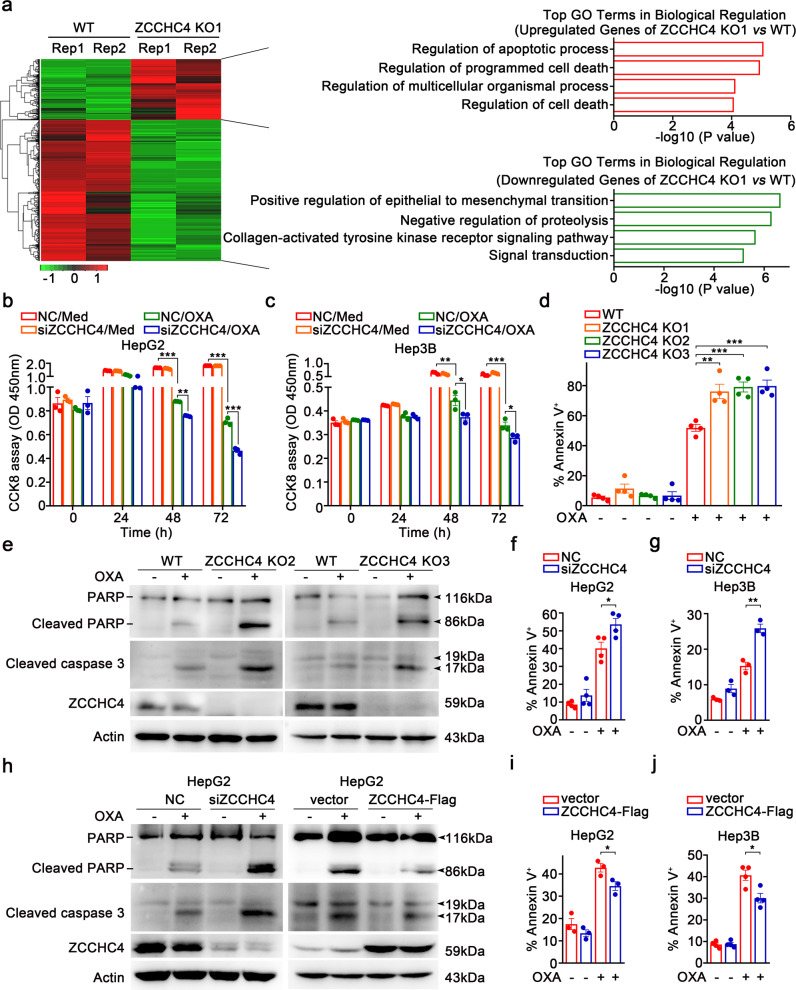
*ZCCHC4* deficiency sensitizes HCC cells to oxaliplatin-induced apoptosis in vitro. **a** RNA-seq analysis of DEGs (*P* < 0.05, false discovery rate <0.001, fold change ≥2) between ZCCHC4 KO1 cells and WT cells. Heatmap (left) and GO analysis of upregulated or downregulated DEGs (right) were shown. DEG differentially expressed genes, WT wild-type, GO gene ontology. **b**, **c** CCK8 assay showing the viability of NC- or *ZCCHC4*- silenced HepG2 cells (**b**) or Hep3B cells (**c**) treated with OXA (12.5 µM) for indicated hours. CCK8 cell counting kit 8, NC non-specific siRNA control. One representative experiment of three is shown. **d** Flow cytometry analyzing OXA (62.5 µM, 36 h)-induced apoptosis in WT cells and ZCCHC4 KO cells (*n* = 4 per group). **e** Immunoblot analysis of OXA (62.5 µM, 16 h)-induced cleaved PARP and cleaved caspase 3 levels in WT cells and ZCCHC4 KO cells. **f**, **g** Flow cytometry analyzing OXA (62.5 µM, 36 h and 62.5 µM, 48 h for HepG2 cells and Hep3B cells respectively)-induced apoptosis in NC- or *ZCCHC4*- silenced HepG2 cells (**f**) or Hep3B cells (**g**) (*n* = 4 and *n* = 3 per group for (**f**) and (**g**), respectively). **h** Immunoblot analysis of OXA (62.5 µM, 16 h)-induced cleaved PARP and cleaved caspase 3 levels in HepG2 cells with or without *ZCCHC4* silence or overexpression. ZCCHC4-Flag, Flag-tagged ZCCHC4-expressing vector. **i**, **j** Flow cytometry analyzing OXA (62.5 µM, 36 h and 62.5 µM, 48 h for HepG2 cells and Hep3B cells, respectively)-induced apoptosis in empty vector- or ZCCHC4-Flag-transfected HepG2 cells (**i**) or Hep3B cells (**j**) (*n* = 3 and *n* = 4 per group for (**i**) and (**j**), respectively). **b**–**d**, **f**, **g**, **i**, **j** Data are shown as mean ± s.e.m. *P < 0.05; **P < 0.01; ***P < 0.001 (unpaired Student’s *t* test). **e**, **h** One representative experiment of three is shown

In order to investigate the mechanisms for ZCCHC4 in promoting HCC growth, we performed RNA-sequencing (RNA-seq) analysis of ZCCHC4 KO1 cells as a primary screening method. *ZCCHC4* knockout resulted in differential expression of a number of genes, including 392 upregulated genes and 926 downregulated genes. Gene ontology analysis indicated that the downregulated genes were enriched in “regulation of epithelial to mesenchymal transition” while the upregulated genes were mainly involved in the regulation of cell death (especially apoptosis) (Fig. 2a), suggesting that ZCCHC4 may regulate the tumorigenic activities of HCC through affecting migration, invasion, and apoptosis of HCC cells.

Further assays indicated that knockout of *ZCCHC4* could significantly inhibit the migration and invasion of WT cells (Supplementary Fig. S3a, b). Moreover, *ZCCHC4* knockdown in HepG2 cells displayed similar effects (Supplementary Fig. S3c–e). Though silence of *ZCCHC4* did not affect HCC proliferation under normal condition, it significantly enhanced OXA-induced cell death of HepG2 cells and Hep3B cells (Fig. 2b, c). Likewise, the silence of *ZCCHC4* promoted OXA-induced inhibition of clonal growth of HepG2 cells and Hep3B cells (Supplementary Fig. S3f, g).

Furthermore, knocking out *ZCCHC4* significantly promoted OXA-induced apoptosis of WT cells (Fig. 2d and Supplementary Fig. S4a), accompanied by increased apoptosis-associated signals, such as cleaved caspase 3 and cleaved PARP (Fig. 2e and Supplementary Fig. S4b). Correspondingly, overexpressing *ZCCHC4* inhibited OXA-induced apoptosis-associated signals in ZCCHC4 KO cells (Supplementary Fig. S4c). Similarly, silence of *ZCCHC4* promoted OXA-induced apoptosis of HepG2 cells and Hep3B cells (Fig. 2f–h and Supplementary Fig. S4d), whereas overexpression of *ZCCHC4* inhibited OXA-induced apoptosis (Fig. 2h–j and Supplementary Fig. S4e). When stimulating HepG2 cells with another DDA, doxorubicin (DOX), ZCCHC4 exerted a similar function in hampering DOX-induced apoptosis and apoptosis-associated signals (Supplementary Fig. S5a–d). In addition to HCC cells, silencing *ZCCHC4* could also promote OXA-induced apoptosis in BXPC-3 PC cells, HCT116 CC cells, and A549 LC cells (Supplementary Fig. S6a–g). These data suggest that ZCCHC4 may promote chemotherapeutic resistance of various cancer cells to DDAs potentially through apoptosis inhibition.

### ZCCHC4 promotes chemoresistance of HCC in vivo

**Fig. 3:**
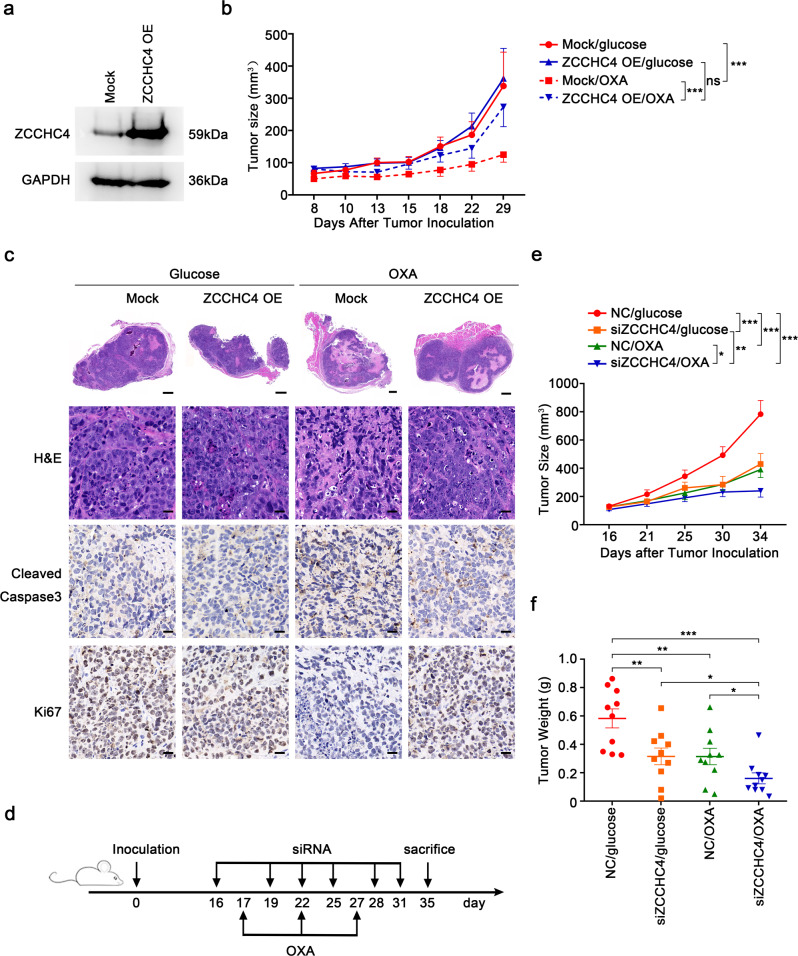
ZCCHC4 hampers oxaliplatin treatment efficiency in HCC in vivo. **a** Representative immunoblot analysis of ZCCHC4 protein in mock cells and cells stably overexpressing ZCCHC4 (ZCCHC4 OE). **b**, **c** Nude mice were inoculated subcutaneously with 1 × 10^7^ ZCCHC4-overexpressing or mock cells and intraperitoneally injected with 10 mg/kg OXA or 5% glucose once a week for three times since day 10 after tumor inoculation (day 0). Tumor growth of mice (*n* = 7 for mice bearing mock cells with 5% glucose treatment or OXA treatment; *n* = 12 and *n* = 10 for mice bearing ZCCHC4 OE cells with 5% glucose treatment or OXA treatment respectively; pooled from two independent in vivo experiments) (**b**) and representative H&E and IHC photographs of tumor tissues (scale bars in top row, 500 μm; scale bars for others, 20 μm) (**c**) were shown. **b** ***P < 0.001; ns, not significant (two-way ANOVA test). **d**–**f** Nude mice were inoculated subcutaneously with 1 × 10^7^ HepG2 cells and received intratumor injection of siRNA (10 nM each mouse) and peritoneal injection of OXA (5 mg/kg) 16 days after tumor inoculation. The scheme was shown in **d**, and the tumor growth (**e**) and tumor weight (**f**) of mice (*n* = 10 for each group, pooled from three independent in vivo experiments) were measured. *P < 0.05; **P < 0.01; ***P < 0.001 (**e** two-way ANOVA test; **f** unpaired Student’s *t* test)

To confirm the roles of ZCCHC4 in HCC in vivo, HepG2 cells stably overexpressing ZCCHC4 (ZCCHC4 OE cells) were constructed using lentivirus (Fig. 3a), and overexpression efficiency was validated in tumor xenografts of nude mice (Supplementary Fig. S7a). We found that OXA treatment could significantly inhibit tumor growth in mice bearing mock cells rather than mice bearing ZCCHC4 OE cells, and overexpression of ZCCHC4 could attenuate OXA-induced tumor growth in vivo (Fig. 3b). Accordingly, less cell death and decreased levels of cleaved caspase 3 in tumor tissues of mice bearing ZCCHC4 OE cells were observed (Fig. 3c and Supplementary Fig. S7b, c). These results confirm the chemoresistance-promoting role of ZCCHC4 in HCC-bearing mice.

### ZCCHC4 knockdown in HCC improves the antitumor effect of OXA in vivo

Next, we intratumorally injected cholesterol-conjugated siRNAs targeting *ZCCHC4* and also intraperitoneally injected OXA into HCC-bearing mice following the schedule shown in Fig. 3d. We found that the combined treatment of *ZCCHC4-*siRNA plus OXA exhibited the most significant antitumor effects, accompanied with the most significantly reduced tumor growth, compared with in vivo administration of siRNA targeting *ZCCHC4* alone or OXA alone (Fig. 3e, f and Supplementary Fig. S7d). So, targeting *ZCCHC4* could increase the sensitivity of HCC to OXA treatment in vivo.

### Restrained DNA damage by ZCCHC4 contributes to chemoresistance of HCC

**Fig. 4:**
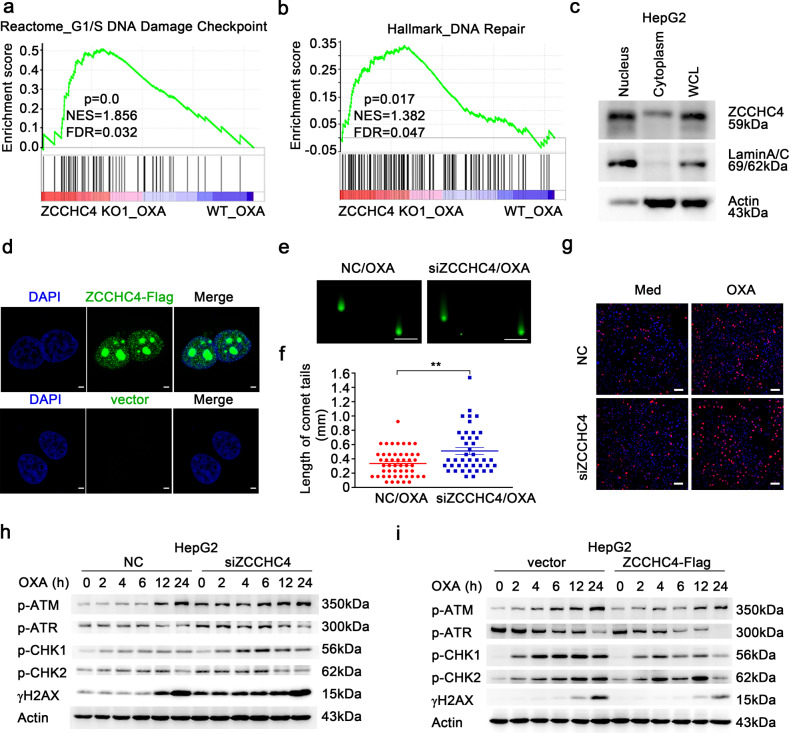
ZCCHC4 restrains oxaliplatin-induced DNA damage. **a**, **b** GSEA analysis of ZCCHC4 KO1 cells and WT cells with OXA (62.5 µM) treatment for 12 h. Reactome (**a**) and hallmark database (**b**) were referred. **c** Representative immunoblot analysis of ZCCHC4 expression in the nucleus and cytoplasm of HepG2 cells. **d** Representative immunofluorescence images (scale bar = 2.5 µm) for ZCCHC4 expression (green) in ZCCHC4-Flag or empty vector-transfected HepG2 cells. ZCCHC4-Flag, Flag-tagged ZCCHC4-expressing vector. **e**, **f** Comet assay for NC- or *ZCCHC4*-silenced HepG2 cells with OXA (62.5 µM) stimulation for 12 h. Representative images (scale bar = 1 mm) (**e**) and lengths of comet tails (*n* = 50 and *n* = 42 for NC and siZCCHC4 group, respectively) (**f**) were shown. **P < 0.01 as determined by unpaired Student’s *t* test (**f**). NC non-specific siRNA control. **g** Representative immunofluorescence images (scale bar = 75 µm) showing γH2AX expression in NC- or *ZCCHC4*- silenced HepG2 cells with OXA (62.5 µM) stimulation for 6 h. **h**, **i** Immunoblot analysis of phosphorylated ATM, ATR, CHK1, CHK2, H2AX levels in HepG2 cells with or without *ZCCHC4* silence (**h**) or overexpression (**i**) followed by OXA (62.5 µM) stimulation for indicated hours. One representative experiment of three is shown

To understand why ZCCHC4 could promote chemoresistance to OXA, we conducted gene set enrichment analysis (GSEA) to define gene sets that were preferentially regulated by ZCCHC4 in response to OXA stimulation. We found that genes correlated with poor survival of HCC patients were minimally expressed in ZCCHC4 KO cells under OXA stimulation (Supplementary Fig. S8a). Besides, genes that were downregulated with high dose ultraviolet C (UVC) irradiation, were much lower in ZCCHC4 KO cells in response to OXA (Supplementary Fig. S8b). UVC predominantly causes DNA damage of cells, and these downregulated genes had been implicated in persistent cell cycle arrest and apoptosis.^26^ Moreover, G1/S DNA-damage checkpoint gene set and DNA repair gene set, two important gene sets related to DNA-damage response (DDR), were highly expressed in ZCCHC4 KO cells in response to OXA (Fig. 4a, b). Considering the GSEA analysis results and that platinum-based drug could form DNA adducts in cells, followed by inducing DDR and ultimately apoptosis^27,28^, we speculated that ZCCHC4 could modulate DDA-induced DDR in HCC cells which may contribute to ZCCHC4-potentiated chemoresistance. ZCCHC4 was mainly localized in the nucleus, where DDR started to take place (Fig. 4c, d and Supplementary Fig. S8c). Silencing *ZCCHC4* in HepG2 cells or Hep3B cells lengthened OXA-induced comet tails (Fig. 4e, f and Supplementary Fig. S8d). *ZCCHC4* knockdown enhanced the intensity of DNA-damage indicator γH2AX in HepG2 cells (Fig. 4g). Consistently, knocking out *ZCCHC4* increased OXA-induced γH2AX levels (Supplementary Fig. S8e), and overexpressing ZCCHC4 decreased γH2AX expression (Supplementary Fig. S8f). When testing the expression levels of γH2AX in tumor tissues of mice receiving combined use of *ZCCHC4-*siRNA and OXA, we found that combo-treatment markedly increased phosphorylation levels of H2AX in tumors (Supplementary Fig. S8g). Thus, ZCCHC4 inhibited OXA-induced DNA damage.

Then we analyzed DDR signals associated with apoptosis after extensive DNA damage. Active ATM and ATR prominently phosphorylates H2AX and spreads DNA-damage signaling. Following the initial nuclear events, DDR signaling is transduced to cytoplasmic targets and among them, the JNK pathway is proved to be necessary for caspase 3-mediated apoptosis. Moreover, JNK could also phosphorylate H2AX on S139 when cells were subjected to overwhelmed DNA damage.^29–31^ Phosphorylation of ATM, ATR, CHK1, CHK2, and H2AX (p-ATM, p-ATR, p-CHK1, p-CHK2, and γH2AX, respectively) were upregulated in *ZCCHC4*-silenced HepG2 cells in response to OXA and sustained for at least 24 h (Fig. 4h). Besides, these DDR-related signals were downregulated when ZCCHC4 was overexpressed (Fig. 4i). Moreover, phosphorylation of JNK, an important DNA-damage-induced apoptosis-related signal, was also increased with knockdown of *ZCCHC4* (Supplementary Fig. S8h). In addition, we tested the role of ZCCHC4 in apoptosis induced by ionizing radiation, and found that silencing *ZCCHC4* promoted ionizing radiation-induced apoptosis (Supplementary Fig. S8i). Taken together, the results demonstrate that ZCCHC4 restrains OXA-induced DDR signals and inhibits DNA-damage-induced apoptosis.

### ZCCHC4 binds a new lncRNA AL133467.2

Given that ZCCHC4 was reported to be a novel m^6^A methyltransferase with the conserved catalytic motif, “DPPF”^21^, we then examined whether ZCCHC4 inhibited DNA-damage-induced apoptosis by its activity of methyltransferase. To make ZCCHC4 catalytically inactive, we mutated “DPPF” to “DAAF”. We found that overexpression of m^6^A methyltransferase-dead ZCCHC4 in ZCCHC4 KO cells could inhibit OXA-induced apoptosis as did by overexpression of ZCCHC4, indicating that ZCCHC4-mediated m^6^A modification did not affect DNA-damage-induced apoptosis (Supplementary S9a, b). Therefore, we speculated that ZCCHC4-mediated inhibition of DDR depended on an alternative manner. ZCCHC4 is an RBP^23^, and long noncoding RNAs (lncRNAs) participate in regulating DDR^32,33^. We wondered whether ZCCHC4 could bind lncRNAs to regulate OXA-induced DDR. We performed ultraviolet cross-linking and immunoprecipitation sequencing (UV-CLIP-seq) assays of HepG2 cells transfected with Flag-tagged ZCCHC4-expressing plasmid (ZCCHC4-Flag) and identified 107 intergenic lncRNAs (lincRNAs) interacting with ZCCHC4, and these lincRNAs show similar binding abundance with ZCCHC4 (data not shown). Since ZCCHC4 was mainly localized in the nucleus, we measured the nuclear abundance of the 107 lncRNAs (Supplementary Fig. S10a) and performed RNA immunoprecipitation-quantitative PCR (RIP-qPCR) to verify the interaction of the top 30 lncRNAs with ZCCHC4. 13 lncRNAs were excluded due to the extremely low abundance in RIP samples. Consistent with the result of UV-CLIP, the remaining 17 lncRNAs could bind with ZCCHC4 (Supplementary Fig. S10b).

**Fig. 5:**
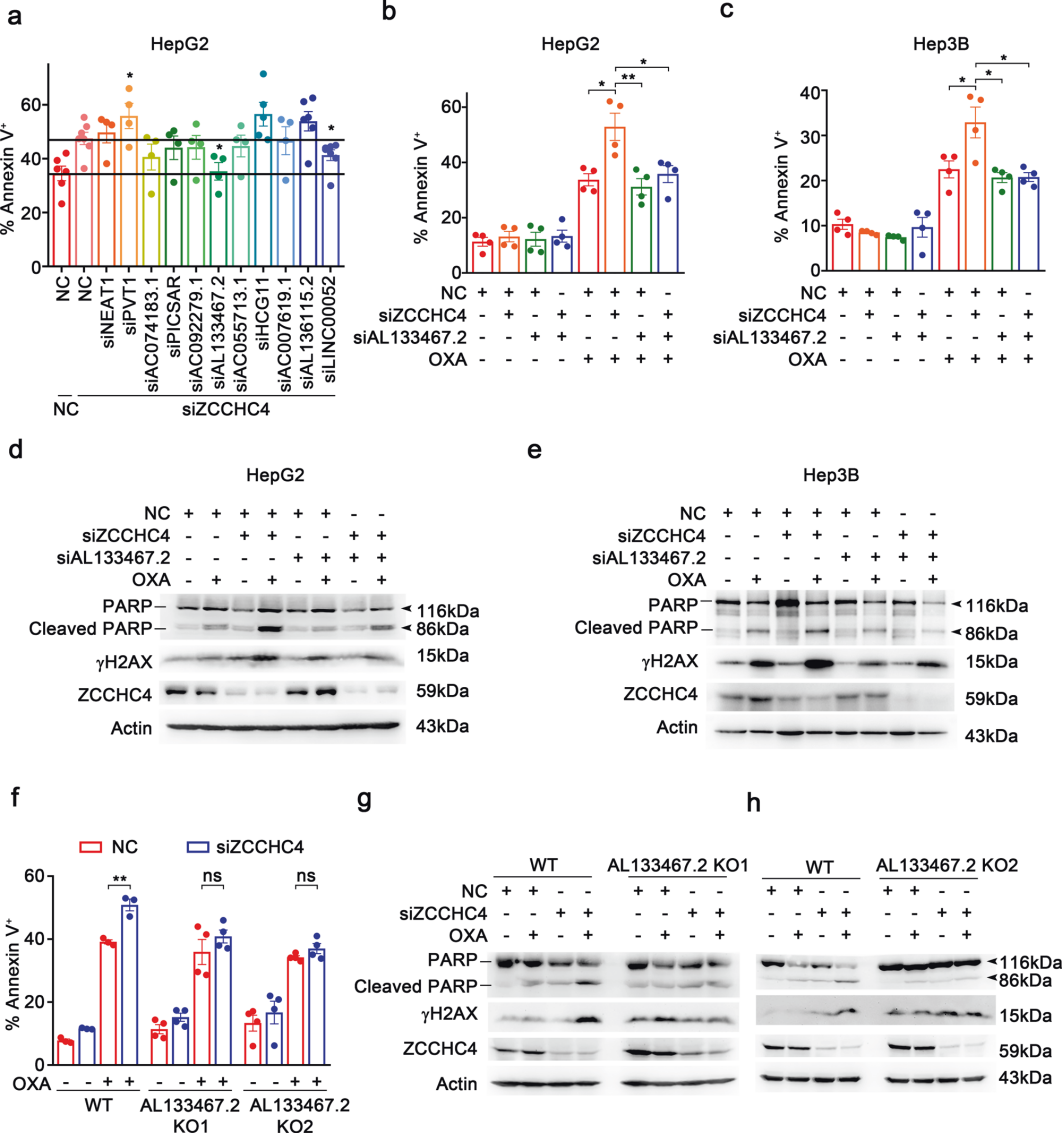
ZCCHC4 hampers the pro-apoptotic function of AL133467.2 in OXA-treated HCC cells. **a** Flow cytometry analyzing OXA (62.5 µM, 36 h)-induced apoptosis of *ZCCHC4*- and lncRNA- co-silenced HepG2 cells (*n* = 4–7 per group). NC, non-specific siRNA control. **b**, **c** Flow cytometry analyzing OXA (62.5 µM, 36 h and 62.5 µM, 48 h for HepG2 cells and Hep3B cells, respectively) -induced apoptosis of *ZCCHC4*- and AL133467.2-co-silenced HepG2 cells (**b**) or Hep3B cells (**c**) (*n* = 4 for (**b**) and (**c**)). **d**, **e** Immunoblot analysis of OXA (62.5 µM, 16 h and 83.3 µM, 18 h for HepG2 cells and Hep3B cells respectively)-induced cleaved PARP and γH2AX levels in *ZCCHC4*- and AL133467.2-co-silenced-silenced HepG2 cells (**d**) and Hep3B cells (**e**). **f** Flow cytometry analyzing OXA (62.5 µM, 36 h)-induced apoptosis of NC- or *ZCCHC4*- silenced WT cells or AL133467.2 KO cells (*n* = 3 and *n* = 4 for WT and AL133467.2 KO group, respectively). WT wild-type. **g**, **h** Immunoblot analysis of OXA (62.5 µM, 16 h) -induced cleaved PARP and γH2AX levels in NC- or *ZCCHC4*- silenced AL133467.2 KO cells. Data are shown as mean ± s.e.m. **a**–**c**, **f** *P < 0.05; **P < 0.01; ns not significant (unpaired Student’s *t* test). **d**, **e**, **g**, **h** One representative experiment of three is shown

Next, smart silencer sequence mixers were constructed to silence these lncRNAs in HCC cells, and 6 of 17 lncRNAs were excluded for non-specificity and low silence efficiency (Supplementary Fig. S10c). Then we examined whether these 11 lncRNAs could regulate the effect of ZCCHC4 and found that silence of lncRNA AL133467.2 could most robustly antagonize the pro-apoptotic effects of *ZCCHC4* silence in HepG2 cells under OXA stimulation (Fig. 5a), indicating that AL133467.2 is functionally associated with ZCCHC4.

As referred to UCSC database, AL133467.2 locates in chromosome 14, and its transcripts are annotated as ENST00000555032.1 or ENST00000662225.1 (Supplementary Fig. S11a). The biological characteristics and function of AL133467.2 has not been reported. Rapid amplification of cDNA ends (RACE) identified that AL133467.2 was a transcript of 1496 nucleotides with a 3’ polyadenylated tail (Supplementary Fig. S11b, c). AL133467.2 had no coding potential, with a PhyloCSF score (<0) as predicted by using NCBI ORF finder (Supplementary Fig. S11d, e). And when cloning AL133467.2 into any frame of pcDNA3.1/Myc expression vector, these vectors did not generate Myc-tagged peptides (Supplementary Fig. S11f). Absolute copy number analysis revealed that AL133467.2 displayed about 48 transcript copies per HepG2 cell (Supplementary Fig. S11g). AL133467.2 located in both the nucleus and cytoplasm (Supplementary Fig. S11h), and OXA stimulation did not obviously change the subcellular location of AL133467.2 in HepG2 cells (Supplementary Fig. S11i). Therefore, AL133467.2 is indeed a ZCCHC4-associated lncRNA that localized in both the nucleus and the cytoplasm.

### ZCCHC4 hampers the pro-DNA-damage and pro-apoptotic function of AL133467.2

We then determined the roles of ZCCHC4-interacting AL133467.2 in chemoresistance. We found that silence of AL133467.2 reversed the effects of *ZCCHC4* knockdown on OXA-induced apoptosis in HCC cells (Fig. 5b, c). Moreover, siAL133467.2 decreased the levels of cleaved PARP and γH2AX under OXA stimulation in ZCCHC4 knockdown HCC cells (Fig. 5d, e). In addition, silence of AL133467.2 alone could not significantly inhibit OXA-induced DNA damage and apoptosis in HCC cells (Fig. 5b–e). So, we speculated that AL133467.2 could promote DNA-damage-induced apoptosis in HCC cells in response to OXA when *ZCCHC4* was deficient. As expected, silence of AL133467.2 in ZCCHC4 KO cells inhibited OXA-induced apoptosis (Supplementary Fig. S12a), as well as the expression of cleaved PARP and γH2AX (Supplementary Fig. S12b–d). Furthermore, overexpression of AL133467.2 in ZCCHC4 OE cells could block the effects of ZCCHC4 overexpression (Supplementary Fig. S12e, f).

To further figure out whether the pro-apoptotic function of *ZCCHC4* deficiency required AL133467.2, we knocked out AL133467.2 in HepG2 cells, and obtained two cell lines (AL133467.2 KO1 cells and KO2 cells), both of which were homozygous knockout (Supplementary Fig. S12g, h). We found that silence of *ZCCHC4* could not promote apoptosis of AL133467.2 KO cells (Fig. 5f and Supplementary Fig. S12i), and also did not increase the levels of cleaved PARP and γH2AX in AL133467.2 KO cells (Fig. 5g, h). So, ZCCHC4 hampers the pro-DNA-damage and pro-apoptotic function of AL133467.2 in HCC cells for promoting chemoresistance to OXA.

### ZCCHC4 and AL133467.2 interact with γH2AX in the nucleus during DNA-damage response

**Fig. 6:**
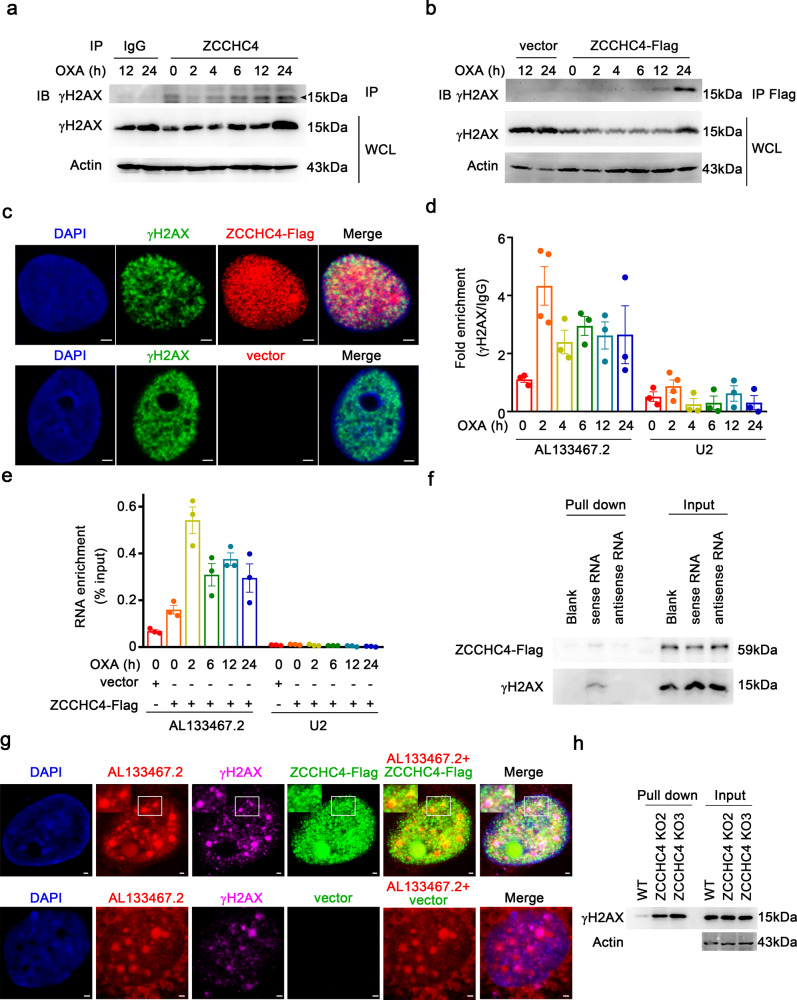
ZCCHC4 and AL133467.2 interact with γH2AX. **a**, **b** Immunoblot analysis of interaction between ZCCHC4 and γH2AX. **a** Immunoprecipitation of γH2AX in HepG2 cells with OXA (62.5 µM) treatment for indicated hours was performed with isotype IgG or ZCCHC4 antibody. **b** Immunoprecipitation of γH2AX in empty vector or ZCCHC4-Flag transfected HepG2 cells with OXA (62.5 µM) treatment for indicated hours was performed with anti-flag magnetic beads. ZCCHC4-Flag, Flag-tagged ZCCHC4-expressing vector. **c** Representative immunofluorescence images (scale bar = 2.5 µm) of Flag (red) and γH2AX (green) in ZCCHC4-Flag or empty vector-transfected HepG2 cells with OXA (62.5 µM) treatment for 24 h. **d**, **e** RIP-assay (**d**) or UV-RIP-assay (**e**) of AL133467.2 enrichment in HepG2 cells with OXA (62.5 µM) treatment for indicated hours. **d** RIP was performed with γH2AX antibody (*n* = 3-4 per group). **e** RIP was performed with anti-flag magnetic beads in empty vector or ZCCHC4-Flag transfected HepG2 cells (*n* = 3 per group). Data are shown as mean ± s.e.m. **f** Immunoblot analysis of ZCCHC4 and γH2AX pulled down by biotin-labeled AL133467.2 in ZCCHC4-Flag transfected HepG2 cells with OXA (62.5 µM) treatment for 12 h. **g** Representative immunofluorescence images (scale bar = 1 µm) of AL133467.2 (red), Flag (green), and γH2AX (magenta) in ZCCHC4-Flag or empty vector-transfected HepG2 cells with OXA (62.5 µM) treatment for 12 h (with white box region magnified; top left corner). **h** Immunoblot analysis of γH2AX pulled down by biotin-labeled AL133467.2 in WT cells or ZCCHC4 KO cells with OXA (62.5 µM) treatment for 12 h. WT wild-type. **a**–**c**, **f**–**h** One representative experiment of three is shown

As to how ZCCHC4 interaction with AL133467.2 inhibited OXA-induced DDR, we wondered whether they interplayed with DDR-associated proteins. Mass spectrometry analysis indicated the interaction between ZCCHC4 and H2AX in response to OXA (Supplementary Fig. S13a). Then, we performed endogenous and exogenous co-immunoprecipitation assays, and found that ZCCHC4 could bind to γH2AX and the binding became more significant along with the treatment time of OXA (Fig. 6a, b). Moreover, the immunofluorescence assay indicated the colocalization of ZCCHC4 and γH2AX in HepG2 cells after OXA stimulation (Fig. 6c and Supplementary Fig. S13b).

Since ZCCHC4 could bind with AL133467.2 and γH2AX, we wondered whether AL133467.2 could bind with γH2AX or ZCCHC4 under OXA treatment. RIP-qPCR or UV-RIP-qPCR analysis showed that AL133467.2 could interact with γH2AX or ZCCHC4 upon the stimulation of OXA, and the binding intensity was most significant when treated with OXA for 2 h (Fig. 6d, e). Considering that ZCCHC4 could bind with γH2AX or AL133467.2 and AL133467.2 could bind with γH2AX under OXA treatment, we asked whether these three components could form a complex. RNA pull-down confirmed that AL133467.2 could simultaneously interact with ZCCHC4 and γH2AX after stimulation of OXA (Fig. 6f), and the three compositions colocalized in the nucleus of HepG2 cells after OXA stimulation (Fig. 6g and Supplementary Fig. S13c, d). Quantification analysis of immunofluorescence images showed that overexpression of ZCCHC4 inhibited the colocalization rate between AL133467.2 and γH2AX (Supplementary Fig. S13d), and RNA pull-down assay further showed that knockout of *ZCCHC4* enhanced the interaction between AL133467.2 and γH2AX (Fig. 6h). These results demonstrated that ZCCHC4 and AL133467.2 dynamically interact with γH2AX to form a nuclear complex in OXA-induced DDR, and ZCCHC4 weakens the interaction between AL133467.2 and γH2AX to promote chemoresistance to DDAs.

Then we investigated which domain of ZCCHC4 was important in promoting chemoresistance. By examining the role of ZCCHC4 protein truncations including fragment 1 (containing Znf-GRF domain), fragment 2 (containing N6-adenineMlase domain), and fragment 3 (containing Znf-CCHC domain) (Supplementary Fig. S13e, f), RIP-qPCR showed that fragment 3 rather than fragment 1 and fragment 2 of ZCCHC4 could bind AL133467.2 (Supplementary Fig. S13g). In addition, overexpression of fragment 3, rather than fragment 1 and fragment 2, of ZCCHC4 in ZCCHC4 KO cells could inhibit OXA-induced apoptosis (Supplementary Fig. S13h). Together, ZCCHC4 functionally interacts with AL133467.2 depending on its Znf-CCHC domain in promoting chemoresistance of HepG2 cells to OXA.

## Discussion

Recent studies have implicated the roles of RBPs in cancer progression and treatment. Therefore, the identification of more RBPs and their partners responsible for chemoresistance will contribute to the development of new therapeutics for cancer patients. Here, we revealed an important role for a rarely studied RBP--ZCCHC4 in promoting HCC chemoresistance, and identified ZCCHC4 as a new predictor of cancer poor prognosis and a potential target for improving chemotherapy effects in cancer patients. Moreover, we demonstrated that the interaction of ZCCHC4 with previously uncharacterized lncRNA AL133467.2 and DNA-damage indicator γH2AX inhibited DNA-damage-induced apoptosis in HCC cells, thus adding new insight to the roles of RBPs in cancer development and chemoresistance.

ZCCHC4 was previously predicted as a potential RNA MTase according to bioinformatic analysis in UniProt Website (UniProt accession number Q9H5U6), which has been verified by recent reports that ZCCHC4 is able to mediate m^6^A methylation of 28S rRNA.^21–23^ Yet, the biological function of ZCCHC4 remains largely unknown. Ma et al. discovered that ZCCHC4 protein is upregulated in the tumor tissue of HCC patients, accompanied by increased m^6^A levels in 28S rRNA. ZCCHC4 could promote the proliferation of HCC cells in vitro and in vivo, which is partially dependent on catalyzing rRNA methylation and promoting optimal global translation.^21^ However, the role of ZCCHC4 in regulating other aspects of cancer biology and pathology, the relationship between ZCCHC4 and cancer prognosis, and the feasibility of ZCCHC4 serving as cancer therapeutic target remain unknown. Also, the detailed signaling pathways influenced by ZCCHC4 in modulating tumor development and therapeutic response are not addressed. In the current study, we comprehensively investigated the biological role of ZCCHC4 in human cancer and revealed that ZCCHC4 could predict the poor prognosis and impede chemotherapy responsiveness of cancer patients with different cancer types. We found a previously unidentified role of ZCCHC4 in tumor chemoresistance, in a manner dependent on inhibition of DNA-damage-induced apoptosis, and that targeting ZCCHC4 could increase chemosensitivity of HCC. In addition to regulating tumor cell apoptosis, whether ZCCHC4 also plays a role in the crosstalk between tumor cells and immune cells in the microenvironment is an intriguing issue to be addressed in the future.

Currently, combination therapy has been widely used to overcome drug resistance in clinical cancer treatment, including chemotherapy, radiotherapy, immunotherapy, and targeted therapy.^34,35^ Targeting epigenetic molecules is emerging as a promising strategy to specifically improve cancer therapy effectiveness^36^. Targeting of RBP and its noncoding RNA partner has been proven to be a potential strategy to overcome drug resistance in HCC^37^. Antisense oligonucleotides (ASOs) and small molecule compound are main strategies for targeting RBPs, whose efficiency and operability have been validated by many preclinical and clinical researches.^38,39^ Our study showed that high expression of ZCCHC4 was related with shorter survival in multiple cancer types and that intratumoral targeting *ZCCHC4* could significantly improve the antitumor effect of chemotherapy in mice bearing inoculated HCC. We thus propose that ZCCHC4 may serve as a predictor for chemotherapeutic sensitivity as well as a potential target in-clinic combination therapy.

The regulators of apoptosis signaling represent attractive avenues for the development of intervention strategies for pathologies where apoptosis is defective, especially cancer.^40^ The response to DNA damage is critical for DDA-induced cell apoptosis. DDR not only participates in DNA repair to remove and tolerate DNA lesion, but also triggers apoptosis to eliminate cells when unrepaired toxic damage occurs.^29,41^ Recent studies have reported that RBPs which localize in the nucleus could interact with proteins or RNAs to participate in DDR regulation, thus influencing the fate of cells.^42,43^ Besides, RBPs and their partners in the nucleus are also important for the stabilty of nuclear structure and genome.^44^ We found that RBP ZCCHC4 could bind with an uncharacterized lncRNA AL133467.2, and dynamically interact with γH2AX to form a triple complex in the nucleus to downregulate the intensity of DNA damage in HCC cells and promote chemoresistance of HCC cells, thus providing new mechanisms of RBP and its interacting molecules in regulation of tumor development and chemoresistance. γH2AX plays a key role in DDR by serving as a docking site for DNA repair machinery which is necessary for cell survival or apoptosis signaling after DNA damage.^29^ Therefore, proper regulation of γH2AX foci is crucial for DDR.^45,46^ Our findings firstly demonstrated that lncRNA AL133467.2 directly interacts with γH2AX which broadens the knowledge about regulatory network of γH2AX interactomes. When DNA damage is overwhelmed by application of DDAs, disrupting the interaction between ZCCHC4 and AL133467.2 will effectively enhance the cancer cells response to chemotherapeutic practice, or even immunotherapeutic practice, considering DDR has also emerged as an important determinant of tumor immunogenicity.^47^

Our findings exhibited that ZCCHC4 could bind a newly identified lncRNA AL133467.2. To note, ZCCHC4 has been reported to only affect the m^6^A modification of rRNA, but not mRNA and lncRNA.^22^ Because ZCCHC4 with m^6^A domain catalytically inactive could still promote chemoresistance and knockdown or depletion of AL133467.2 could largely rescue the effect of *ZCCHC4* deficiency, we inferred that ZCCHC4-mediated chemoresistance was mainly due to the interaction between ZCCHC4 and AL133467.2, but not via its m6A methyltransferase activity. We also demonstrated that ZCCHC4 bound AL133467.2 mainly depending on Znf-CCHC domain rather than m^6^A methyltransferase domain, and Znf-CCHC fragment phenocopied the function of full-length ZCCHC4 in OXA-induced apoptosis, further suggesting that ZCCHC4-mediated chemoresistance results from its interplay with lncRNA.

Moreover, the underlying mechanisms about how ZCCHC4, AL133467.2, and γH2AX interplay during the process of DDR and how ZCCHC4 and AL133467.2 affect the phosphorylation of H2AX are also worthy of further investigations. Since PI3K kinases are important for phosphorylation of H2AX, and p-ATM signal, one important PI3K kinase, is critical for the antitumor effect of OXA^48,49^, it is interesting to figure out whether ZCCHC4 and AL133467.2 could regulate the recruitment of p-ATM to affect γH2AX signal. Crystal structure of human ZCCHC4 displayed that flanking Znf domains packed around the MTase domain, cooperating to form an integrated RNA-binding platform in ZCCHC4 protein.^50^ Further investigation will be needed to find out the more critical domains or sites involving in the interaction of ZCCHC4-AL133467.2-γH2AX complex which will do favor to uncovering more action mode of ZCCHC4.

## Materials and methods

### Ethics approval statements

All animal experiments were conducted in accordance with the National Institute of Health Guide for Care and Use of Laboratory Animals, with the approval of the Scientific Investigation Board of Second Military Medical University (Shanghai, China).

### Animals and cell lines

Male nude mice (6–8 weeks) were purchased from Joint Ventures Sipper BK Experimental Animal (Shanghai, China). HepG2 cell and BXPC-3 cell were purchased from Cell Resource Center of the Chinese Academy of Science (Shanghai, China); Hep3B cell was from FuHeng Cell Center (Shanghai, China); HCT116 cell, A549 cell, and HEK293T cell were from ATCC (Manassas, VA, USA). Cells were cultured as suggested, and authenticated using STR profiling. ZCCHC4 KO cell and AL133467.2 KO cell were constructed by CRISPR/Cas9 technology as previously reported^18^, and HepG2 cell stably overexpressing ZCCHC4 were constructed by using lentiviral vector.

### Tissue microarray and IHC

TMAs (HCC, HLiv-HCC180Sur05; pancreatic cancer, HPanAde170Sur01; colon cancer, HColA180Su11; lung cancer, HLugA180Su05.) were purchased from Outdo Biotech (Shanghai, China). Patients who lacked paired non-tumor tissues or had tissue flaking or failed to follow-up were excluded. IHC staining was performed as previously described.^51^ ZCCHC4 antibody (ab154002) was used as the primary antibody.

### Antibodies and reagents

The antibodies and reagents used in this study are listed in Supplementary Table S1.

### Immunoprecipitation

HepG2 cells were treated with 62.5 µM OXA for indicated time, harvested, sonicated, and immunoprecipitated with anti-Flag M2 Magnetic Beads for 7 h or antibody specific to ZCCHC4 overnight following protein A agrose beads inoculation for 3 h. The beads were washed by NTEN buffer (1 mM EDTA, 0.5% NP40, 20 mM Tris-HCl, pH 8.0) from high-saline solution to low-saline solution for three times.

### RNA immunoprecipitation (RIP) and UV-RIP

Cells were treated with 1% formaldehyde (Thermo Fisher) for 10 min, 0.125 M glycine for 5 min. Then cells were dissolved with RNase-free lysis buffer and sonicated on ice. Supernatants were inoculated with anti-Flag M2 Magnetic Beads for 7 h or antibodies specific to γH2AX overnight following protein A agarose beads inoculation for 3 h. The beads were washed with RIP buffer (150 mM KCl, 25 mM Tris pH 7.4, 5 mM EDTA, 0.5% NP40) for three times and then treated with protease K and 1% SDS at 55 °C and shaken for 30 min. RNA was obtained using TRIzol reagent, and analyzed by qRT-PCR.

For UV-RIP, cells were treated with 0.15 J/cm^2^ of 254 nm UV light in a crosslinker HL-2000 (UVP) for cross-linking. The following procedure was similar to RIP.

### UV-CLIP seq

In all, 2 × 10^7^ ZCCHC4-Flag transfected HepG2 cells were subjected to cross-linking with 0.15 J/cm^2^ of 254 nm UV light in a crosslinker HL-2000 (UVP). Cells were harvested and lysed with cell lysis buffer for 1 h at 4 °C to obtain the protein extract. The extract was then treated with RNase T1 (1 U/ml) at 22 °C for 15 min followed by transferring to ice for 5 min. The lysates supplemented with cocktail, phenylmethanesulfonyl fluoride (PMSF) and RNase inhibitor were incubated with anti-Flag M2 Magnetic Beads for 7 h at 4 °C. The beads were washed with NTEN 900 buffer (1 mM EDTA, 0.5% NP40, 20 mM Tris-HCl, pH 8.0, 900 mM NaCl) once and NTEN 300 buffer (1 mM EDTA, 0.5% NP40, 20 mM Tris-HCl, pH 8.0, 300 mM NaCl) twice. The NTEN buffer mentioned was supplemented with cocktail, PMSF, and RNase inhibitor. Then beads were linked with a biotin-labeled L3 linker, and separated on a 4–12% NuPAGE gel (Invitrogen). The protein–RNA complexes were transferred to the nitrocellulose filter membrane and detected by a chemiluminescent kit. The target protein–RNA complexes were cut and digested by Protease K. Finally, RNA was isolated and subjected to high-throughput sequencing using Illumina HiSeq 3000 with SE50 strategy.

### RNA fluorescence in situ hybridization (FISH) and immunofluorescence (IF) microscopy

TAMRA-conjugated AL133467.2 probes for FISH assay were designed by the Stellaris FISH Probe Designer (Biosearch Technologies, http://www.biosearchtech.com/stellaris-designer) and synthesized in Shanghai BiOligo Biotech Co., LTD (China). The probes for AL133467.2 are listed in Supplementary Table S2. IF assay was conducted as previously reported.^52^ HepG2 cells were treated with fixation and permeabilization solution (BD Pharmingen) for 30 min, and hybridization was carried out using AL133467.2 probe sets according to the protocol of Biosearch Technologies. To investigate colocalization, cells were pre-incubated with rabbit anti-flag antibody (Cell Signaling Technology) and mouse anti-γH2AX antibody (Abcam) followed by Alexa Flour 488 anti-rabbit secondary antibody and Cyanine 5 anti-mouse secondary antibody (Thermo Fisher). Images were obtained with confocal microscopy (Leica).

### RNA pull-down assay

Biotin-labeled sense or antisense AL133467.2 were transcribed in vitro using T7 RNA polymerase transcription kit (Promega) and Biotin RNA Labeling Mix (Roche). The isolated biotin-labeled RNA was heated to 95 °C for 2 min and placed on ice for 3 min. The folded RNA was then added to the lysate of ZCCHC4-Flag transfected HepG2 cells with OXA treatment for 12 h, which have been pre-precipitated with streptavidin-coupled beads (Thermo Fisher) at 37 °C for 30 min before, and rotated at 37 °C for 1 h. Then, 60 µl of streptavidin-coupled beads (Thermo Fisher) were added to the reaction and rotated at 37 °C for 1 h. After washing with RIP buffer from high-saline solution to low-saline solution four times, the beads were separated by SDS-PAGE followed by immunoblot analysis.

### Statistical analysis

The experiments for animal models were independently repeated at least twice, and other experiments were independently repeated at least three times. SPSS 19.0 and Graphpad Prism 8.0 were used for data analysis. The expression of ZCCHC4 examined by IHC in patients was shown as median, and other results were shown as mean ± s.e.m. Statistical analysis of survival data was performed by the Kaplan–Meier method and analyzed by the Log-rank test. The significance of comparisons between two groups was determined by the Student’s *t* test and comparison of more than two groups determined by ANOVA test. The expression of ZCCHC4 in tumor and paired non-tumor tissues were analyzed using the paired, two-tailed Student’s *t* test and others using unpaired, two-tailed Student’s *t* test. *P* values < 0.05 was considered to be statistically significant.

## Supplementary information

Supplementary materials

## Data Availability

All data supporting the findings of this study are included in the article and/or the supplementary materials. The original data sets are also available from the corresponding author upon request. The RNA-sequencing data from this study are deposited in NCBI GEO under accession code GSE188704, which contains three subseries GSE188702, GSE188703, and GSE190632.

## References

[1] Gebauer F, Schwarzl T, Valcárcel J, Hentze MW (2021). RNA-binding proteins in human genetic disease. Nat. Rev. Genet..

[2] Choi PS, Thomas-Tikhonenko A (2021). RNA-binding proteins of COSMIC importance in cancer. J. Clin. Investig..

[3] Fabbri L, Chakraborty A, Robert C, Vagner S (2021). The plasticity of mRNA translation during cancer progression and therapy resistance. Nat. Rev. Cancer.

[4] Duffy AG (2016). Modulation of tumor eIF4E by antisense inhibition: A phase I/II translational clinical trial of ISIS 183750-an antisense oligonucleotide against eIF4E-in combination with irinotecan in solid tumors and irinotecan-refractory colorectal cancer. Int. J. Cancer.

[5] Shen L, Pelletier J (2020). Selective targeting of the DEAD-box RNA helicase eukaryotic initiation factor (eIF) 4A by natural products. Nat. Prod. Rep..

[6] Bowling EA (2021). Spliceosome-targeted therapies trigger an antiviral immune response in triple-negative breast cancer. Cell.

[7] Ishak CA, Loo Yau H, De Carvalho DD (2021). Spliceosome-targeted therapies induce dsRNA responses. Immunity.

[8] Xu Y (2021). ERα is an RNA-binding protein sustaining tumor cell survival and drug resistance. Cell.

[9] Su R, Qing Y, Chen J (2021). Targeting differentiation blockade in AML: new hope from cell-surface-based CRISPR screens. Cell Stem Cell.

[10] Wang E (2021). Surface antigen-guided CRISPR screens identify regulators of myeloid leukemia differentiation. Cell Stem Cell.

[11] Vasan N, Baselga J, Hyman DM (2019). A view on drug resistance in cancer. Nature.

[12] Sung H (2021). Global cancer statistics 2020: GLOBOCAN estimates of incidence and mortality worldwide for 36 cancers in 185 countries. CA Cancer J. Clin..

[13] Wei X (2021). MiR-125b loss activated HIF1α/pAKT loop, leading to transarterial chemoembolization resistance in hepatocellular carcinoma. Hepatology.

[14] Bosc C, Selak MA, Sarry JE (2017). Resistance is futile: targeting mitochondrial energetics and metabolism to overcome drug resistance in cancer treatment. Cell Metab..

[15] Cabral LKD, Tiribelli C, Sukowati CHC (2020). Sorafenib resistance in hepatocellular carcinoma: the relevance of genetic heterogeneity. Cancers.

[16] Liu A (2020). A novel strategy for the diagnosis, prognosis, treatment, and chemoresistance of hepatocellular carcinoma: DNA methylation. Med Res Rev..

[17] Li GH (2021). Super-enhancers: a new frontier for epigenetic modifiers in cancer chemoresistance. J. Exp. Clin. Cancer Res.

[18] Chen K (2017). Methyltransferase SETD2-mediated methylation of STAT1 is critical for interferon antiviral activity. Cell.

[19] Budhwani M, Mazzieri R, Dolcetti R (2018). Plasticity of type I interferon-mediated responses in cancer therapy: from anti-tumor immunity to resistance. Front Oncol..

[20] Ma L (2021). LSD1-demethylated LINC01134 confers oxaliplatin resistance via SP1-induced p62 transcription in hepatocellular carcinoma. Hepatology.

[21] Ma H (2019). N(6-)Methyladenosine methyltransferase ZCCHC4 mediates ribosomal RNA methylation. Nat. Chem. Biol..

[22] van Tran N (2019). The human 18S rRNA m6A methyltransferase METTL5 is stabilized by TRMT112. Nucleic Acids Res..

[23] Pinto R (2020). The human methyltransferase ZCCHC4 catalyses N6-methyladenosine modification of 28S ribosomal RNA. Nucleic Acids Res..

[24] Zhang Z (2021). m(6)A regulators as predictive biomarkers for chemotherapy benefit and potential therapeutic targets for overcoming chemotherapy resistance in small-cell lung cancer. J. Hematol. Oncol..

[25] Tang Z, Li C, Kang B, Gao G, Zhang Z (2017). GEPIA: a web server for cancer and normal gene expression profiling and interactive analyses. Nucleic Acids Res..

[26] Gentile M, Latonen L, Laiho M (2003). Cell cycle arrest and apoptosis provoked by UV radiation-induced DNA damage are transcriptionally highly divergent responses. Nucleic Acids Res..

[27] Yuan X (2020). Proteomic analysis of cisplatin- and oxaliplatin-induced phosphorylation in proteins bound to Pt-DNA adducts. Metallomics.

[28] Combès E (2019). Inhibition of ataxia-telangiectasia mutated and RAD3-related (ATR) overcomes oxaliplatin resistance and promotes antitumor immunity in colorectal cancer. Cancer Res..

[29] Roos WP, Thomas AD, Kaina B (2016). DNA damage and the balance between survival and death in cancer biology. Nat. Rev. Cancer.

[30] Picco V, Pagès G (2013). Linking JNK activity to the DNA damage response. Genes Cancer.

[31] Podhorecka M, Skladanowski A, Bozko P (2010). H2AX phosphorylation: its role in DNA damage response and cancer therapy. J. Nucleic Acids.

[32] Barcena-Varela M, Lujambio A (2021). A novel long noncoding RNA finetunes the DNA damage response in hepatocellular carcinoma. Cancer Res..

[33] Michelini F (2017). Damage-induced lncRNAs control the DNA damage response through interaction with DDRNAs at individual double-strand breaks. Nat. Cell Biol..

[34] Zhang C, Bollag G (2021). Triple therapy to outwit the BRAF oncogene. Cancer Discov..

[35] Huang A, Yang XR, Chung WY, Dennison AR, Zhou J (2020). Targeted therapy for hepatocellular carcinoma. Signal Transduct. Target Ther..

[36] Wang Z, Wang B, Cao X (2021). Epigenetic checkpoint blockade: new booster for immunotherapy. Signal Transduct. Target Ther..

[37] Xu J (2020). CircRNA-SORE mediates sorafenib resistance in hepatocellular carcinoma by stabilizing YBX1. Signal Transduct. Target Ther..

[38] Lewin J (2018). Phase Ib trial with birabresib, a small-molecule inhibitor of bromodomain and extraterminal proteins, in patients with selected advanced solid tumors. J. Clin. Oncol..

[39] Hong D (2015). AZD9150, a next-generation antisense oligonucleotide inhibitor of STAT3 with early evidence of clinical activity in lymphoma and lung cancer. Sci. Transl. Med..

[40] Sekhar SC (2019). A H2AX^-^CARP-1 interaction regulates apoptosis signaling following DNA damage. Cancers.

[41] Bouwman P, Jonkers J (2012). The effects of deregulated DNA damage signalling on cancer chemotherapy response and resistance. Nat. Rev. Cancer.

[42] Bampton A, Gittings LM, Fratta P, Lashley T, Gatt A (2020). The role of hnRNPs in frontotemporal dementia and amyotrophic lateral sclerosis. Acta Neuropathol..

[43] Vohhodina J (2021). BRCA1 binds TERRA RNA and suppresses R-Loop-based telomeric DNA damage. Nat. Commun..

[44] Wang X (2021). Mutual dependency between lncRNA LETN and protein NPM1 in controlling the nucleolar structure and functions sustaining cell proliferation. Cell Res..

[45] Lee JH (2017). ID3 regulates the MDC1-mediated DNA damage response in order to maintain genome stability. Nat. Commun..

[46] Nowsheen S (2018). ZNF506-dependent positive feedback loop regulates H2AX signaling after DNA damage. Nat. Commun..

[47] Chabanon RM (2021). Targeting the DNA damage response in immuno-oncology: developments and opportunities. Nat. Rev. Cancer.

[48] Hawley BR, Lu WT, Wilczynska A, Bushell M (2017). The emerging role of RNAs in DNA damage repair. Cell Death Differ..

[49] Wu Q (2020). Hsa_circ_0001546 acts as a miRNA-421 sponge to inhibit the chemoresistance of gastric cancer cells via ATM/Chk2/p53-dependent pathway. Biochem Biophys. Res Commun..

[50] Ren W (2019). Structure and regulation of ZCCHC4 in m(6)A-methylation of 28S rRNA. Nat. Commun..

[51] Han Y (2018). Tumor-induced generation of splenic erythroblast-like Ter-cells promotes tumor progression. Cell.

[52] Yu Z (2021). TRIM41 is required to innate antiviral response by polyubiquitinating BCL10 and recruiting NEMO. Signal Transduct. Target Ther..

