# Supplementary material for: RNA-binding protein ZCCHC4 promotes human cancer chemoresistance by disrupting DNA-damage-induced apoptosis
Source: Signal Transduct Target Ther. 2022 Jul 20;7:240. doi: 10.1038/s41392-022-01033-8 (PMC9296561; doi:10.1038/s41392-022-01033-8)
Supplement: Supplementary file 1 — Supplementary materials [file 41392_2022_1033_MOESM1_ESM.docx]

Supplementary Materials for

RNA-binding protein ZCCHC4 promotes human cancer chemoresistance by disrupting DNA-damage-induced apoptosis

Ha Zhu^1^, Kun Chen^2^, Yali Chen^2^, Juan Liu^1^, Xiaomin Zhang^1^, Yumei Zhou^2^, Qiuyan Liu^1^, Bingjing Wang^2^, Taoyong Chen^1^, Xuetao Cao^1,2,3,*^

Correspondence to: [caoxt@immunol.org](mailto:caoxt@immunol.org)

**This PDF file includes:**

Materials and Methods

Figures. S1 to S13

Tables S1 to S2

References

Materials and Methods

*In vivo* Tumor Models

1 × 10^7^ cells were subcutaneously injected into nude mice, and tumor sizes of tumor-bearing mice were measured by using a caliper and calculated as 0.5 × length × width^2^. The survival of tumor-bearing mice was monitored daily. To evaluate the antitumor activity of OXA *in vivo*, mice bearing ZCCHC4-overexpressed or mock cells were intraperitoneally injected with 10 mg/kg OXA or 5% glucose control solution once a week for 3 times since day 10 after tumor inoculation. Mice were sacrificed on day 35 and tumors were resected for H&E and IHC analysis.

To evaluate the antitumor activity of combo treatment of siRNA targeting *ZCCHC4* and OXA *in vivo*, tumor bearing mice were treated as previously described.^1^ 10 nmol cholesterol-conjugated siRNA for *ZCCHC4* or non-specific siRNA control in 50 μl saline solution was intratumorally injected every 3 days for 6 times while 5 mg/kg OXA or 5% glucose control solution was intraperitoneally injected every 5 days for 3 times. Tumor bearing mice were sacrificed on day 35.

TCGA (The Cancer Genome Atlas) Database Analysis

Transcriptome data were derived from TCGA liver hepatocellular carcinoma (LIHC) cohort and analyzed on GEPIA (Gene Expression Profiling Interactive Analysis) (http://gepia.cancer-pku.cn/), a web-based tool to deliver fast and customizable functionalities based on TCGA and GTEx RNA sequencing data.^2^ Expression of *ZCCHC4* and survival plot of patients with high and low *ZCCHC4* levels (third quartile group cutoff) were evaluated.

RNA Sequencing and Analysis

Total RNA from ZCCHC4 KO1 cells and WT cells with or without OXA stimulation was extracted using TRIzol reagent (Invitrogen) according to the manufacturer’s protocol. Each group has two repetitions. cDNA library construction and sequencing were performed by Beijing Genomics Institute using BGISEQ-500 platform. High-quality reads were aligned to the human reference genome (GRCh38) using Bowtie2. The expression levels of genes were normalized to fragments per kilobase of exon model per million mapped reads (FPKM) using RNA-seq by Expectation Maximization (RSEM). GO analysis of the differentially expressed genes were performed on the website of the BGI Genomics Co.,Ltd. (http://report.bgi.com). In addition, gene set enrichment analysis (GSEA) was performed according to the instruction.^3^

Flow Cytometry

For apoptotic cell analysis, cells were treated with 62.5 μM OXA or 2 μM DOX for indicated time (HepG2 cells and BXPC-3 cells, 36 h; Hep3B cells and A549 cells, 48 h; HCT116 cells, 24 h), harvested, washed and stained with Annexin V-APC and SYTOX Green at 4℃ in dark for 30 min. Data were obtained using Fortessa (BD Biosciences) or LSRII (BD Biosciences) and analyzed by Flowjo software.

Cytotoxicity Assay

2 × 10^3^ HepG2 cells or Hep3B cells were seeded into 96-well plates and transfected with siRNAs for 24 h followed by 12.5 μM OXA treatment for indicated time. Then Cell Counting Kit 8 (CCK8) assay was performed following the instruction with CCK8 solution incubated for 2 h at 37℃.

Plate Clone Formation Assay

1 × 10^4^ HepG2 cells or Hep3B cells were seeded into 6-well plates and transfected with siRNAs for 24 h followed by the treatment of 12.5 μM OXA for 2 h. The plates were washed and then incubated with fresh medium every 5 days. 10-14 days later, the cells were fixed with 4% paraformaldehyde and stained with crystal violet solution.

*In Vitro* Migration and Invasion Assay

Migration assay was conducted using wound healing assay, and invasion assay was performed using 24-well Boyden chambers (10 μm thickness and 8 μm pore size) (BD Biosciences, Bedford, MA). 1 × 10^5^ HCC cells in 200 μl FBS-free culture medium were added to the upper chamber and incubated for 24 h, and then filters were harvested, fixed with paraformaldehyde, stained with 0.5% crystal violet and counted under a microscope (Olympus) in 5 pre-determined fields.

Small RNA-mediated Interference

For *ZCCHC4* interference, cancer cells were transfected with siRNAs (GenePharma) using INTERFERin (Polyplus) at a final concentration of 20 nM following manufacturer’s instructions.

For lncRNA interference, Ribo^TM^ Smart Silencer (contain three siRNAs and three antisense oligonucleotides targeting different sequences) for each lncRNA was designed and synthesized in Ribobio (China). Cancer cells were transfected with the mixture of smart silencer and siRNA (1:1) using INTERFERin (Polyplus) at a final concentration of 33.3 nM. The sequence of siRNAs and Smart Silencers were listed in Supplemental Table S2.

Plasmid Constructs and Transfection

The recombinant vector encoding Flag-tagged human *ZCCHC4* was constructed by PCR-based amplification from cDNA of HepG2 cells followed by subcloning into pcDNA3.1/Flag eukaryotic expression vector (Invitrogen). The recombinant vector expressing AL133467.2 was constructed by Rapid Amplification of Cloned cDNA Ends (RACE) and subcloned into pcDNA3.1/Myc eukaryotic expression vector (Invitrogen). Cancer cells were transfected with recombinant vectors using JetPEI reagents (PolyPlus) according to the manufacturer’s instructions.

Immunoblot Analysis

To test the DNA-damage-associated and apoptosis-associated signal levels, HepG2 cells were transfected with siRNA or recombinant vectors followed by 62.5 μM OXA or 2 μM DOX treatment for 16 h or indicated time. Hep3B cells were transfected with siRNA followed by 83.3 μM OXA treatment for 18 h. Cells or frozen tumor tissues were lysed via cell lysis buffer (Cell Signaling Technology) supplemented with protease inhibitor cocktail (Calbiochem) and the protein concentrations were determined via bicinchoninic acid assay (Pierce). Equalized extracts were used for immunoblot analysis as described previously.^4^ The nucleus and cytoplasm fractions were prepared using NE-PER Nuclear and Cytoplasmic Extraction Reagents (Thermo Fisher) following manufacturer’s instructions supplemented with protease inhibitor cocktail (Calbiochem).

Quantitative Real-Time RT-PCR

Total RNA was extracted from cultured cells with TRIzol Reagent (Invitrogen) or RNA_fast200_ kit (Fastagen) following the manufacturer’s instructions. For subcellular RNA extracts from cells, cells were prepared with NE-PER Nuclear and Cytoplasmic Extraction Reagents (Thermo Fisher) supplied with RNase inhibitor (Sangon Biotech) followed by TRIzol Reagent treatment. qRT-PCR analysis was performed using SYBR RT-PCR kit (Takara) and LightCycler (Roche) as described previously.^5^ qRT-PCR primers used for indicated lncRNAs and mRNAs were listed in Supplemental Table S2.

Neutral Comet Assay

Neutral comet assay was performed using Comet assay kit (Trevigen) following the instruction. NC- or *ZCCHC4*- silenced HCC cells with OXA treatment (HepG2 cells, 62.5 μM OXA, 12 h; Hep3B cells, 83.3 μM OXA, 24h), were harvested in ice cold PBS. Cell suspension was mixed with low-melting agarose (v/v 1:10) and transferred to pre-coated slides. Cells were lysed in neutral lysis solution at 4℃ overnight and then washed with electrophoresis buffer and run for 45 min at 16 V at 4℃. After several washes in ddH_2_O, nuclei were stained with SYBR Green (Takara) (1:2 in TE buffer (10 mM Tris-HCl, 1 mM EDTA, pH 8.0)) at room temperature for 30 min, washed with ddH_2_O and dried at 37℃. Pictures were taken using Olympus microscope.

Rapid Amplification of Cloned cDNA Ends (RACE)

Total RNA extracted from HepG2 cells was subjected to RACE PCR with SMARTer RACE 5’/3’ Kit (Takara) according to the manufacture’s protocol.

AL133467.2 sequence obtained from RACE (5’-3’ 1496nt):

ACATGGGGGCGGAAGTGGGAGCGCGGCAAGCGGATGGAGCCAGCGAGAAACACATAGCCAGGGTTCTGTCGCGTGACCCCCGACGGCACCTGCTCCCCATCAGCAGCATCATACTCCGATGGATAAACTGAGGGCTCAGTGGGTCATGACCTGCCCAGAGTCATGGAGGGAGTGAGAGGCAGAGCCAGGATGAGCTCCCAGGCCCTGAAACTTCCGAGCCAGTGGCCTACACACAGAGCTTCCCACCCTTCCTCATTGGGTTTCTGCAAGCCCTCCCCACCCCAGCACTCTGCAGGGCCTGGGAAGTGAACCAGCTGGGAGGATTCTTAGAGAAGAAAAGTGCTGATTTAAGAGAAACTGCATTATTTGTGAAATACAAAATTCATCTTGTGGGTGTAAGAGATGCCTTCGTGGACGGATGAAGCTCCTGGTACTAAGTAGCAGGAGTGCAGTGAAGCTGCTGTGTTCTCCAGCTCGTAGTTAAGGGCCAGTCAGGATCAGACATCTTGCCCTGATGGAAAAGAAGATGAACAGATACCTCCTGACCTGGATAGCAATTAATTCTGGGGGCCTAGATCCCCAAGGACTTTAGGATGAGGCAGCAGTTTGGATTTAGGATCAACATGGTATTTAAAATATAAAAGCATTGTAGTGTTGGGGAAGCATCATTTAAAAGAACTTGTGGGGTCCCTGTCAGGCCCCTCTTGAGACAGGCCAGCCCCACAGAAGAGAAGGCCTAGGGCTGGCTCCCTAGGGTTGTGAGAAACTCCAGAACAGTGTTGACTTCTTTCTTCCCCTAGGGTCACACCAGCATGTAGCATGATGCCTTGCACACAGTAGGTGCTCAGTAAACACTTTTGGACCACTGAATGCAATGACCAATGGATAAAAACATTGTTCTTCCCTTGACCAGATAAGACCCACTTCCCTGGGCGTCCACAGGCCGCTGGTGATGCACACTCTTCTCTGTGTGGCCTCTGCCTGCCGGGGGAAGCCTTGTGACCAGTTTCTCCCCACTGAGTCAAGGGAGAATGAGGGGGAGCATGAACATTTGCTTGTTTCCCTTATGGCTTCTGAATCTTTATTTTATTTTTTTTAATGAAAATGTCTACTTGAATTTAGGTAAGGAAGACCTTGCTCTTAGTTTTTGAATTTTGAACTAGAAAAAAAAAAAGGTCTTTCAATGAACACTAGCAGAACAGTCTAAGCAATCATGATAAAGACCTAATTCCTGCCTCTTTCTCCTGCTCTGATGGAGCTGATGACAGCACAAATGGGAGAGGGAGAATGTAGCAAGGACACTGAAGGACAGAGAGTTGTCCAAGCCACCAGGAAGGTGAAAGTGGGAATTCATTTTCTGTGCCATTGGGTGGTTCCTTAGGAAAAAATGTAGACAAAGAAAAATGTTGTATTATTTGTGGAAATCAAGTTCGTATTGCAGGTGTAAGAAATGTCTTTGTGGGTTGACAGCTAAAAAAAAAAAAAAAAAAAAAAAA


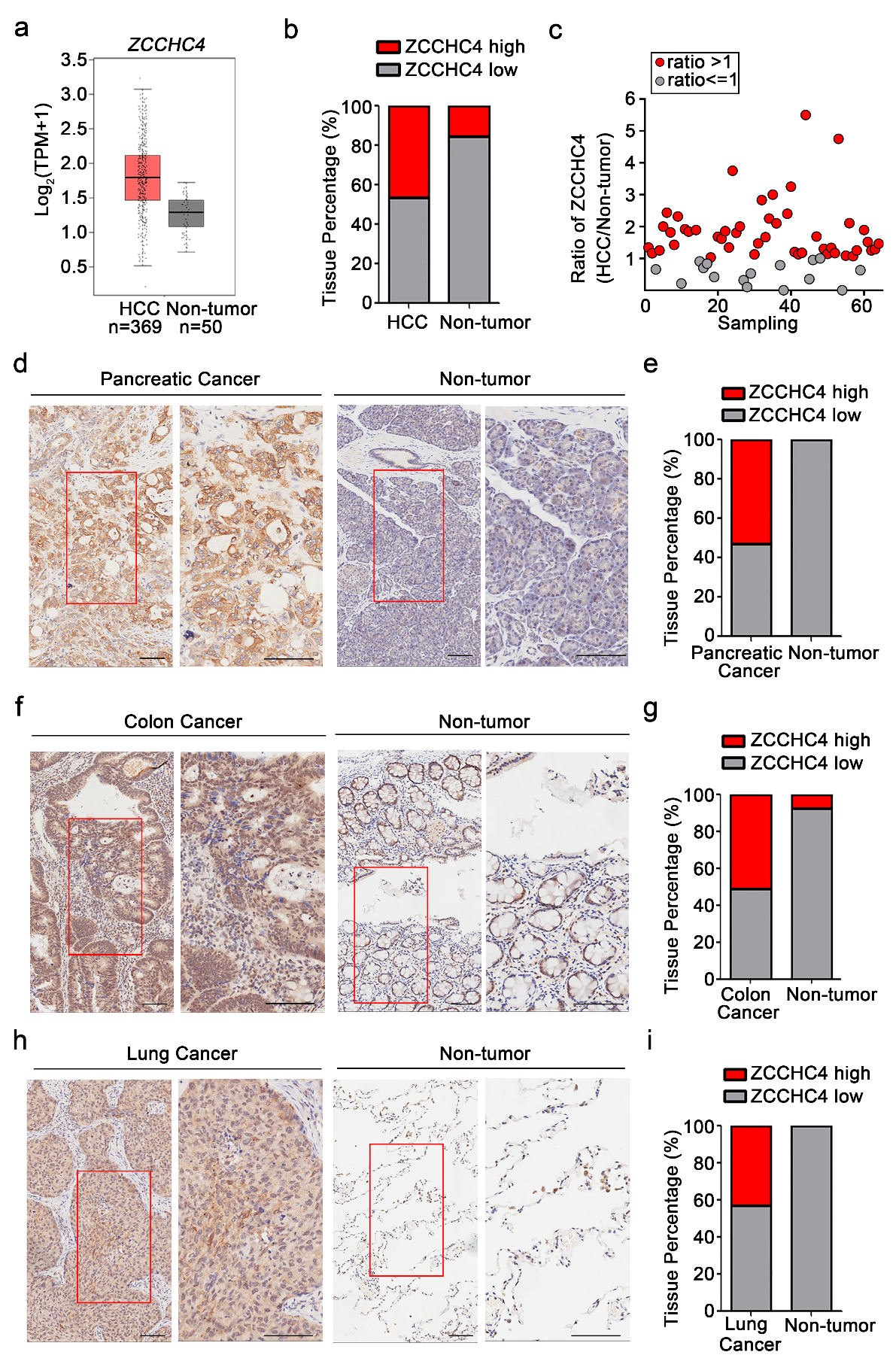


Figure. S1.

**ZCCHC4 level is increased in human cancers. a** *ZCCHC4* mRNA expression (horizontal lines, median level; whiskers, 2.5-97.5 percentile range) in HCC and non-tumor tissues analyzed in GEPIA. TCGA normal data as non-tumor tissue; TPM, transcripts per million mapped reads. **b** Percentage of high and low expression of ZCCHC4 (median in tumor tissue as group cutoff) in HCC patients (n = 64). **c** Ratios of ZCCHC4 expression in HCC tissues compared to paired non-tumor tissues of 64 patients. **(d-i)** (d, f, h) Representative IHC images (scale bar = 100 μm) of ZCCHC4 protein detection in pancreatic cancer patients (d), or colon cancer patients (f) or lung cancer patients (h) (with red box region magnified; right). (e, g, i) Percentage of high and low expression of ZCCHC4 (median in tumor tissue as group cutoff) in pancreatic cancer patients (n = 68) (e), colon cancer patients (n = 80) (g) and lung cancer patients (n = 83) (i).


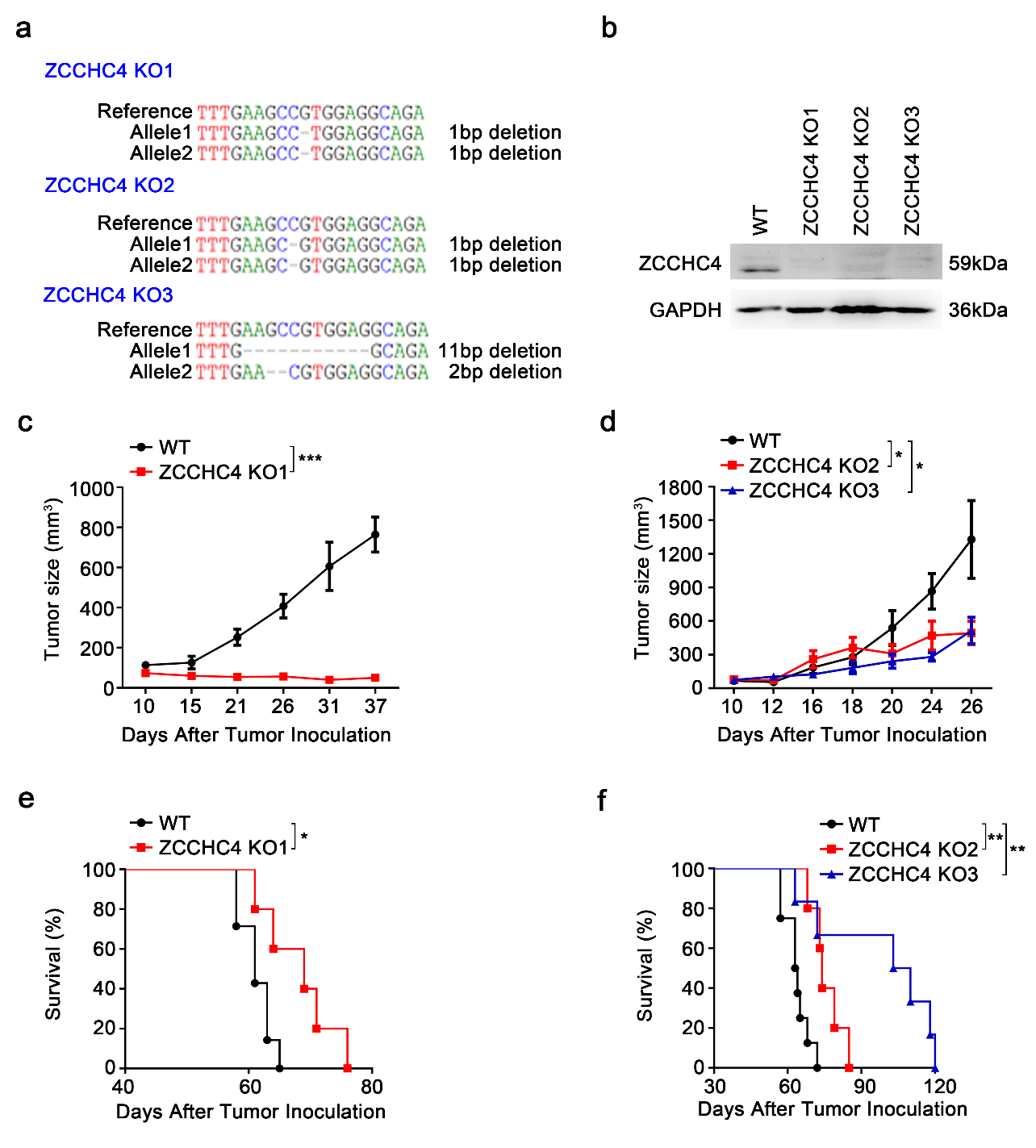


Figure. S2.

**ZCCHC4 deficiency inhibits HCC growth *in vivo* and prolongs the survival of tumor-bearing mice. a** Sanger-sequencing analysis of genomic DNA of ZCCHC4 KO cells (frameshift mutations). **b** Immunoblot analysis of ZCCHC4 expression in three ZCCHC4 KO cells and WT cells. WT, wild type. One representative experiment of three was shown. **c-f** Tumor growth in nude mice after subcutaneous injection of 1 × 10^7^ ZCCHC4 KO cells and WT cells (c and d) and the Kaplan-Meier survival curves of tumor-bearing mice (e and f). (c and e) n = 7 and n = 5 for mice bearing WT cells and ZCCHC4 KO1 cells respectively. (d and f) n = 8, n = 5 and n = 6 for mice bearing WT cells, ZCCHC4 KO2 cells and ZCCHC4 KO3 cells respectively. *, p < 0.05; **, p < 0.01; ***, p < 0.001 (two-way ANOVA test in c and d; Log-rank test in e and f).


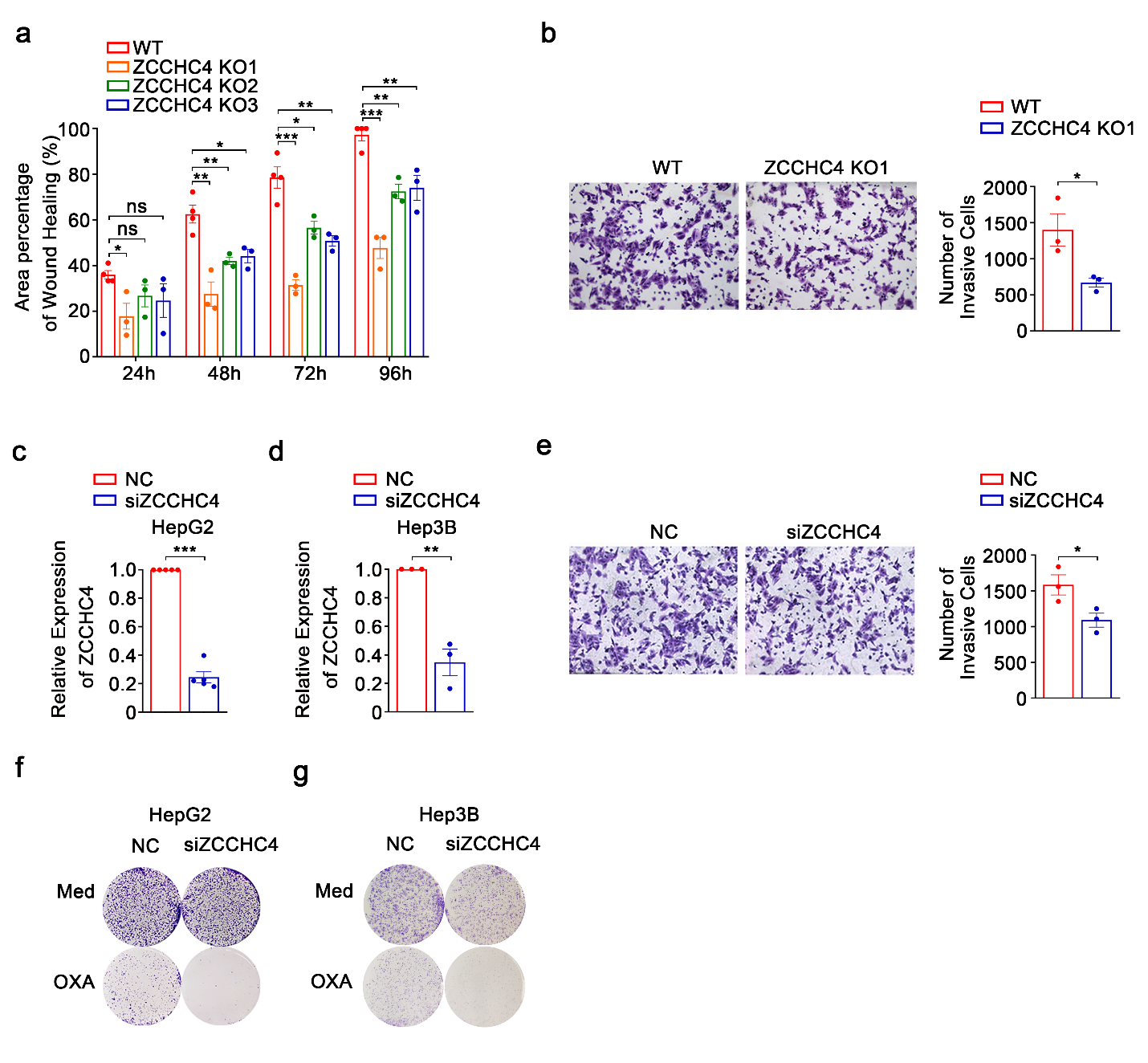


Figure. S3.

**Characterization of ZCCHC4 KO cell. a** Migration assay of WT cell and ZCCHC4 KO cell (n = 4 and n = 3 for WT cell and ZCCHC4 KO cell respectively). **b** Invasion assay of WT cell and ZCCHC4 KO cell (n = 3 per group). Representative images (left) and histogram data (right) were shown. **c, d** qRT-PCR analysis of *ZCCHC4* expression in NC- or *ZCCHC4*- silenced HepG2 cells (c) and Hep3B cells (d) (n = 5 and n = 3 for c and d respectively). NC, non-specific siRNA control. **e** Invasion assay of NC- or *ZCCHC4*- silenced HepG2 cells (n = 3 per group). Representative image (left) and histogram data (right) were shown. **f, g** Plate cloning assay of NC- or *ZCCHC4*- silenced HepG2 cells (f) or Hep3B cells (g) with OXA (12.5 µM) treatment for 2 h. Data were shown as mean ± sem (a-e). *, p < 0.05; **, p < 0.01; ***, p < 0.001; ns, not significant (a-e; unpaired Student’s *t* test).


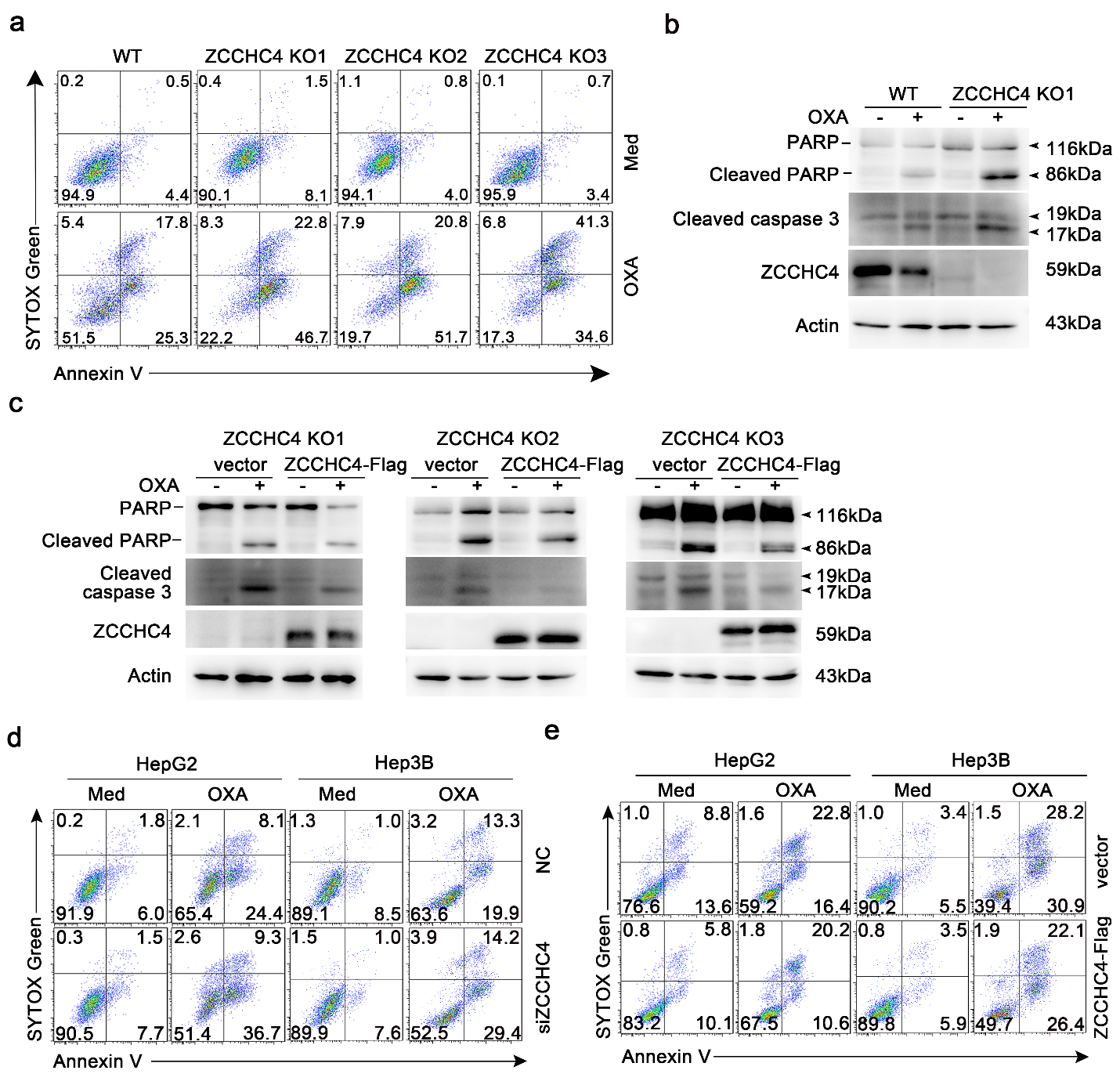


Figure. S4.

**ZCCHC4 deficiency sensitizes HCC cell to oxaliplatin treatment *in vitro*. a** Flow cytometry analyzing OXA (62.5 µM, 36 h) -induced apoptosis in WT cells and ZCCHC4 KO cells. **b** Immunoblot analysis of OXA (62.5 µM, 16 h)-induced cleaved PARP and cleaved caspase 3 levels in WT cells and ZCCHC4 KO1 cells. **c** Immunoblot analysis of OXA (62.5 µM, 16 h) -induced cleaved PARP and cleaved caspase 3 levels in empty vector or ZCCHC4-Flag transfected ZCCHC4 KO cells. ZCCHC4-Flag, Flag-tagged ZCCHC4 expressing vector. **d** Flow cytometry analyzing OXA (62.5 µM, 36 h and 62.5 µM, 48 h for HepG2 cells and Hep3B cells respectively) -induced apoptosis in NC- or *ZCCHC4*- silenced HepG2 cells and Hep3B cells. NC, non-specific siRNA control. **e** Flow cytometry analyzing OXA (62.5 µM, 36 h and 62.5 µM, 48 h for HepG2 cells and Hep3B cells respectively) -induced apoptosis in empty vector or ZCCHC4-Flag transfected HepG2 cells and Hep3B cells. (a-e) One representative experiment of three was shown.


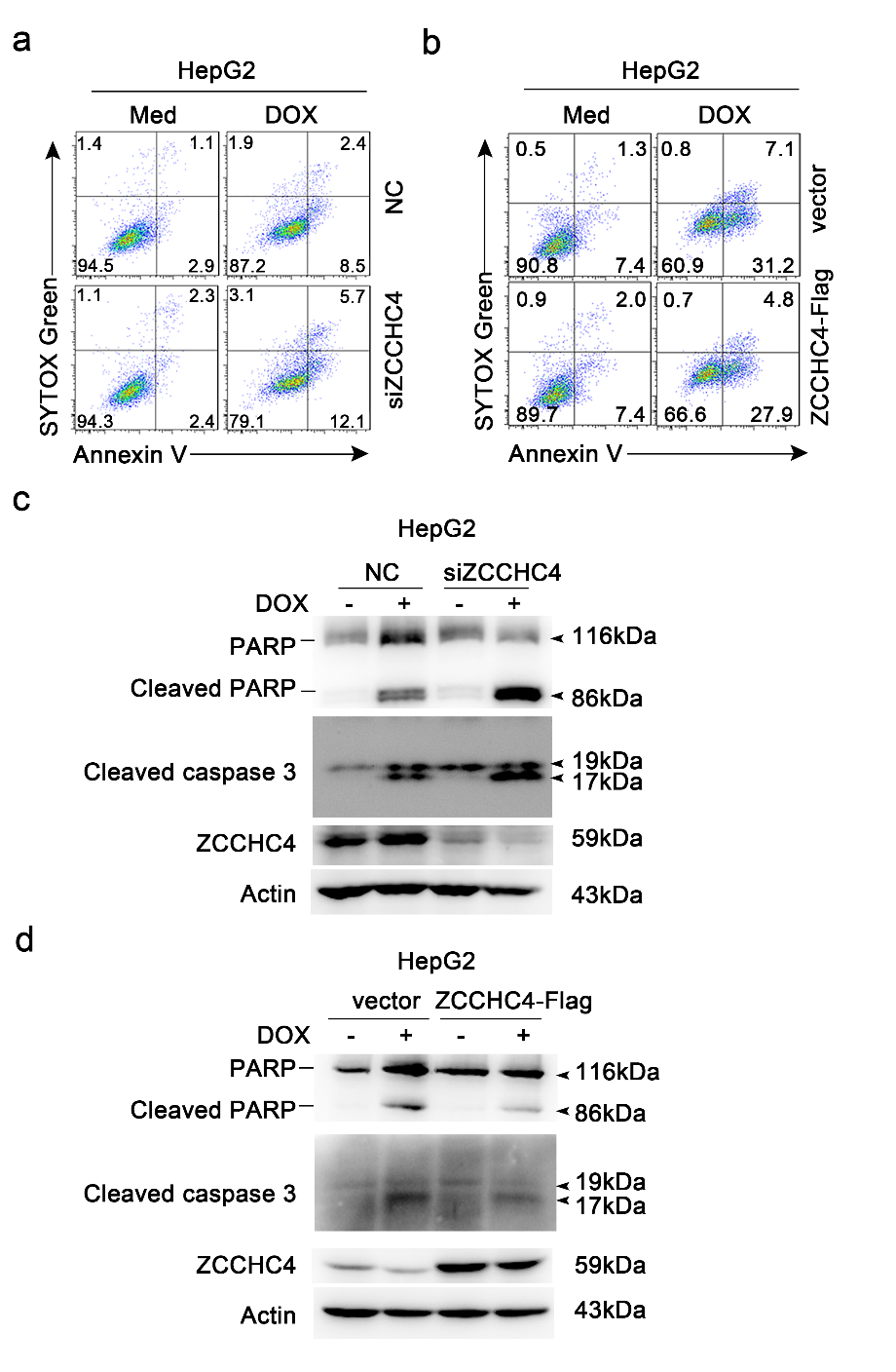


Figure. S5.

**ZCCHC4 deficiency sensitizes HCC cell to doxorubicin treatment *in vitro*. a** Flow cytometry analyzing DOX (2 µM, 36 h) -induced apoptosis in NC- or *ZCCHC4*- silenced HepG2 cells. NC, non-specific siRNA control. **b** Flow cytometry analyzing DOX (2 µM, 36 h) -induced apoptosis in empty vector or ZCCHC4-Flag transfected HepG2 cells. **c** Immunoblot analysis of DOX (2 µM, 16 h) -induced cleaved PARP and cleaved caspase 3 levels in NC- or *ZCCHC4*- silenced HepG2 cells. **d** Immunoblot analysis of DOX (2 µM, 16 h) -induced cleaved PARP and cleaved caspase 3 levels in empty vector or ZCCHC4-Flag transfected HepG2 cells. (a-d) One representative experiment of three was shown.


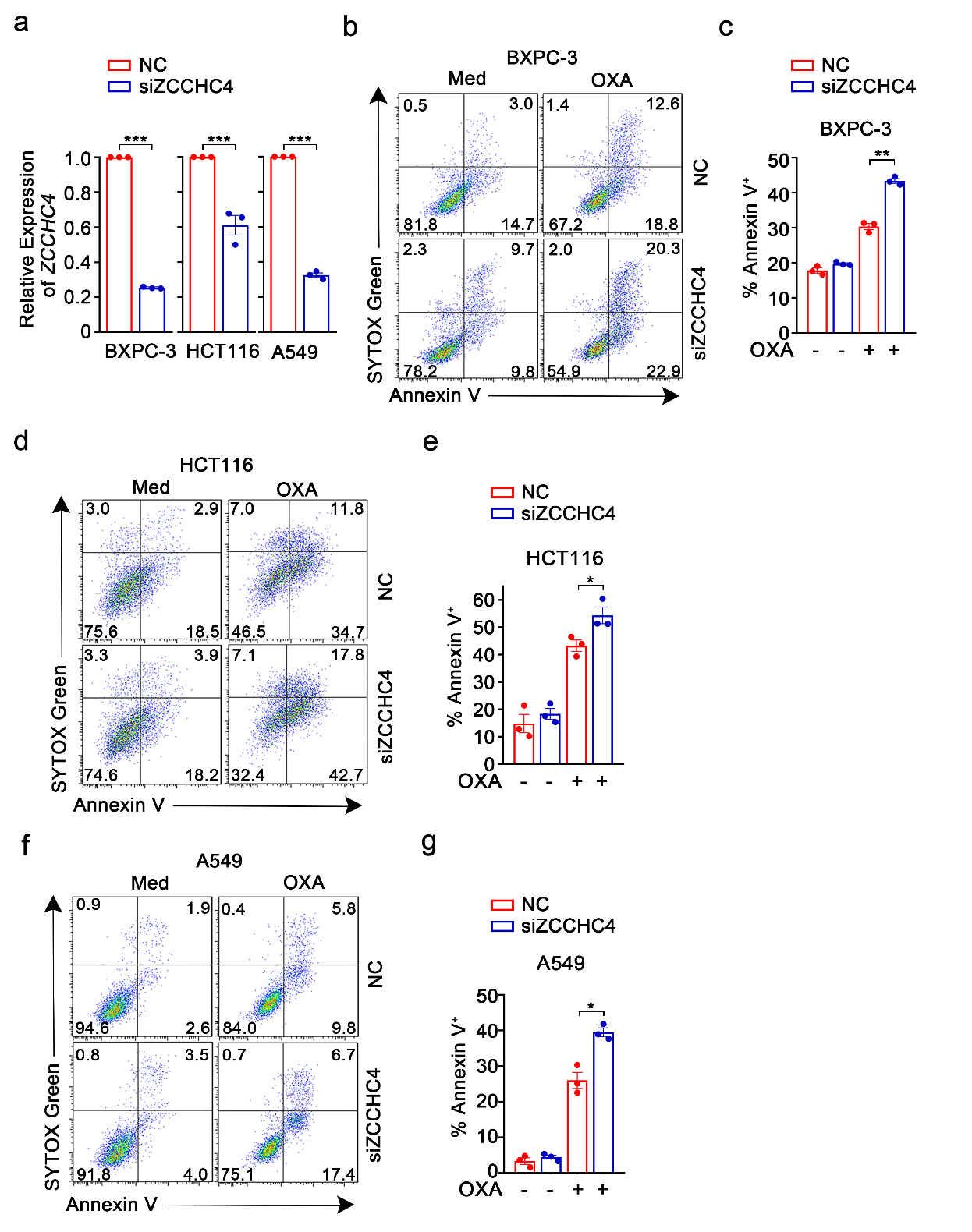


Figure. S6.

**ZCCHC4 promotes chemoresistance of pancreatic, colon and lung cancer cells. a** qRT-PCR analysis of ZCCHC4 expression in NC- or *ZCCHC4*- silenced BXPC-3 cells, HCT116 cells and A549 cells (n = 3 per group). NC, non-specific siRNA control. **b-g** Flow cytometry analyzing apoptosis in NC- or *ZCCHC4*- silenced BXPC-3 cells (b and c), HCT116 cells (d and e) and A549 cells (f and g) with or without OXA (62.5 µM) treatment for indicated time (36 h, 24 h and 48 h for BXPC-3 cells, HCT116 cells and A549 cells respectively) (n = 3 per group). Representative graphs in (b, d, f) and histograms in (c, e, g) were shown. Data were shown as mean ± sem (a, c, e, g). *, p < 0.05; **, p < 0.01; ***, p < 0.001 (unpaired Student’s *t* test).


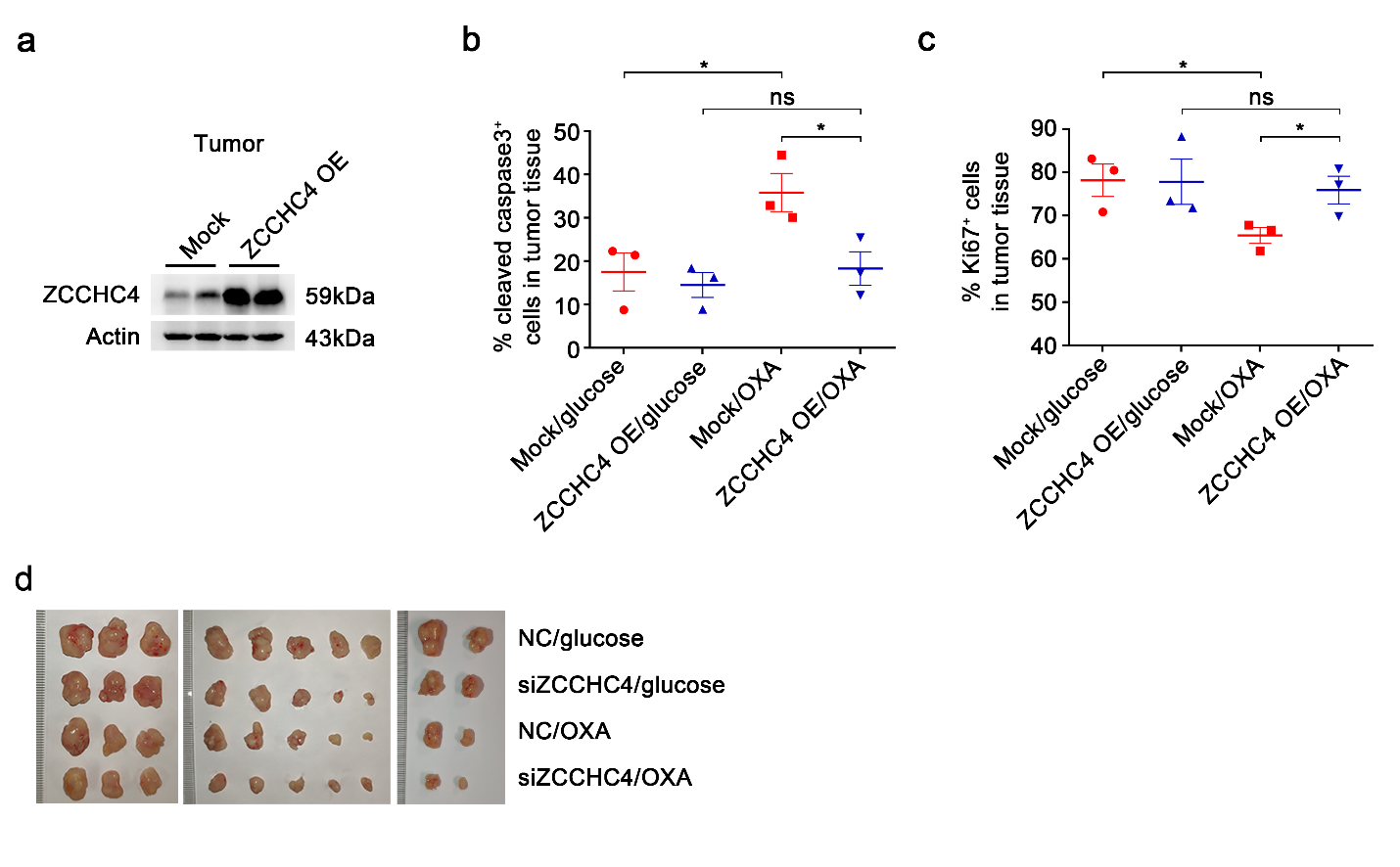


Figure. S7.

**ZCCHC4 deficiency sensitizes HCC cell to oxaliplatin treatment *in vivo*.** **a** Representative Immunoblot analysis of ZCCHC4 expression in tumor tissues of mice bearing mock cells and ZCCHC4 OE cells (n = 2 for each group). ZCCHC4 OE, HepG2 cells stably overexpressing ZCCHC4. **b, c** Nude mice were inoculated subcutaneously with 1 × 10^7^ ZCCHC4-overexpressing or mock cells and intraperitoneally injected with 10 mg/kg OXA or 5% glucose once a week for 3 times since day 10 after tumor inoculation (day 0). Quantifications of cleaved caspase 3 (b) and Ki67 signals (c) in xenografts (n = 3 for each group) were shown. *, p < 0.05; ns, not significant (unpaired Student’s *t* test). **d** The images of resected tumors from sacrificed mice in Fig. 3e (n = 10 for each group, pooled from three independent in vivo experiments).

**
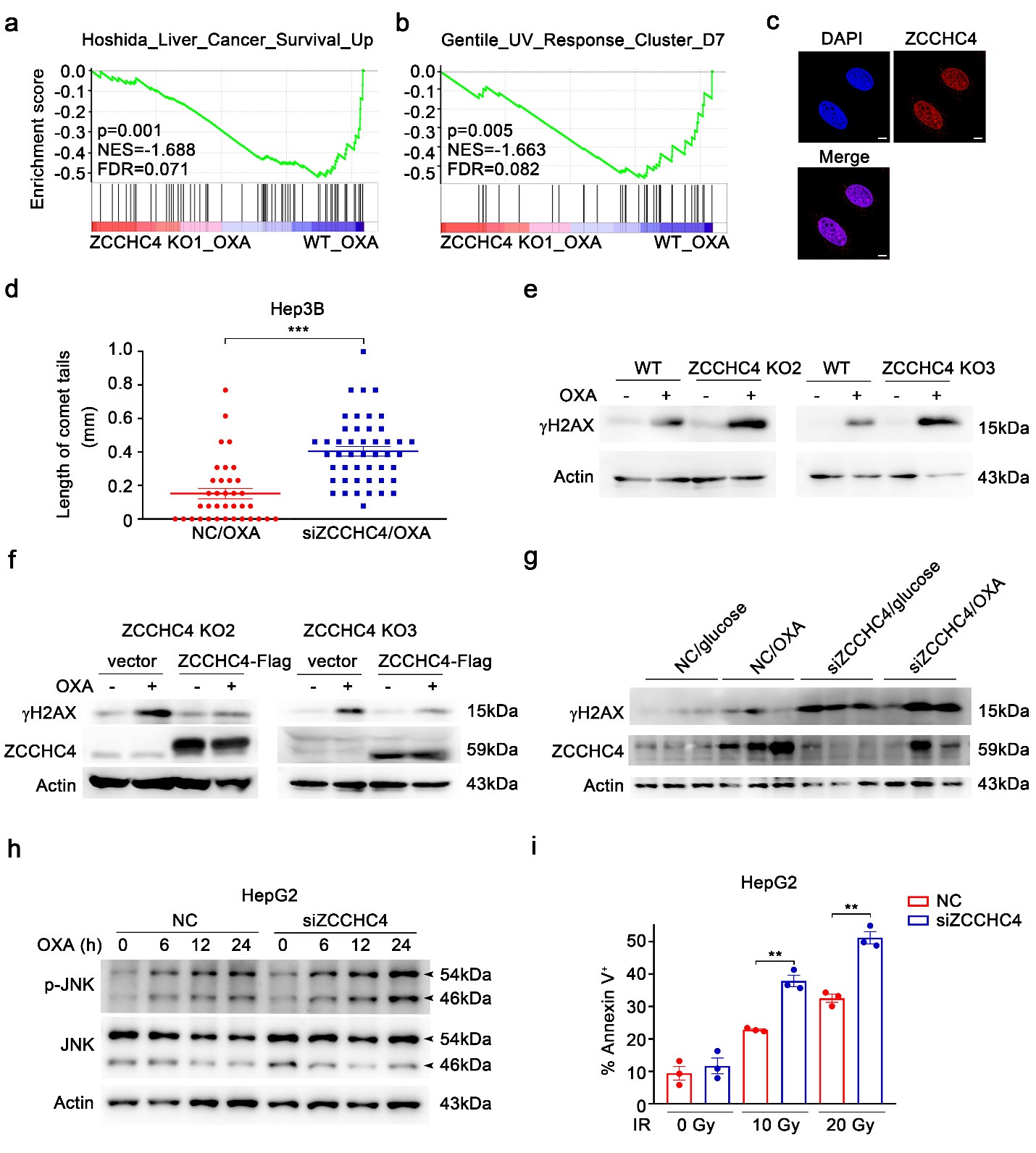
**

Figure. S8.

**ZCCHC4 deficiency promotes OXA-induced DNA damage of HCC cell *in vitro*. a, b** GSEA analysis of ZCCHC4 KO1 cells and WT cells with OXA (62.5 µM) treatment for 12 h. **c** Immunofluorescence images (scale bar = 5 μm) for ZCCHC4 expression (red) in HepG2 cells. **d** Comet assay for NC- or *ZCCHC4*- silenced Hep3B cells with OXA (83.3 µM) stimulation for 24 h. Lengths of comet tails (n = 36 and n = 47 for NC and si*ZCCHC4* group respectively) were shown. NC, non-specific siRNA control. **e** Immunoblot analysis of OXA (62.5 µM, 16 h) -induced γH2AX levels in WT cells and ZCCHC4 KO cells. WT, wild type. **f** Immunoblot analysis of OXA (62.5 µM, 16 h) -induced γH2AX levels in empty vector or ZCCHC4-Flag transfected ZCCHC4 KO cells. ZCCHC4-Flag, Flag-tagged ZCCHC4 expressing vector. **g** Representative immunoblot analysis of ZCCHC4 and γH2AX levels in tumor tissues of mice receiving combined treatment of OXA and siRNA targeting *ZCCHC4* or monotreatment (n = 3 for each group). **h** Immunoblot analysis of phosphorylated JNK and JNK levels in NC- or *ZCCHC4*-silenced HepG2 cells with OXA stimulation (62.5 µM) for indicated time. NC, non-specific siRNA control. **i** Flow cytometry analysis of apoptosis in NC- or *ZCCHC4-* silenced HepG2 cells with or without ionizing radiation (10 Gy or 20 Gy) for 48 h (n = 3 per group). NC, non-specific siRNA control. (c, e-h) One representative experiment of three was shown. Data were shown as mean ± sem (d, i). **, p < 0.01; ***, p < 0.001 (unpaired Student’s *t* test).


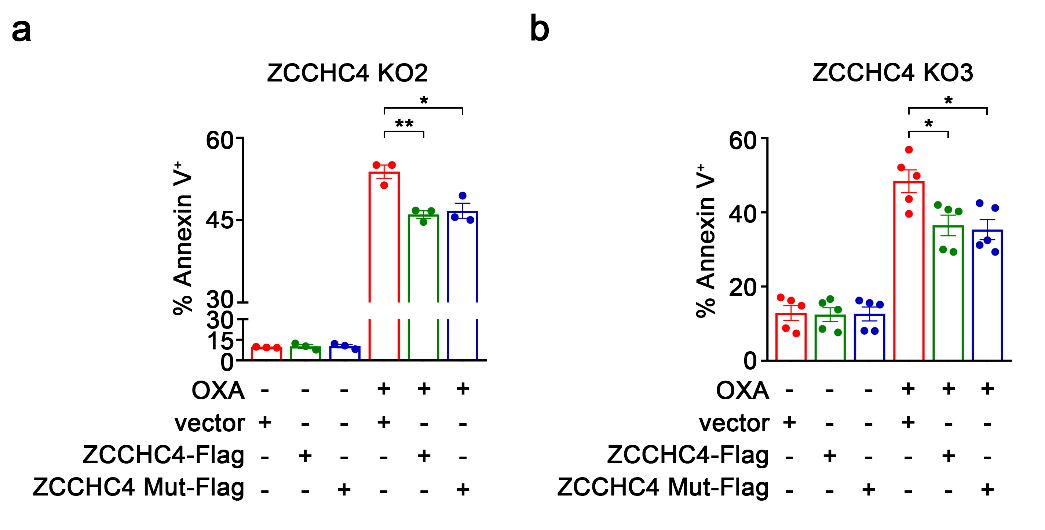


Figure. S9.

**ZCCHC4 with m^6^A domain catalytically inactive still exerts the apoptosis-resistant role in HCC cells. a, b** Flow cytometry analysis of OXA (62.5 µM, 36 h) -induced apoptosis in empty vector or ZCCHC4-Flag or ZCCHC4 Mut-Flag transfected ZCCHC4 KO cells (n = 3 and n = 5 for (a) and (b) respectively). *, p < 0.05; **, p < 0.01 (unpaired Student’s *t* test). ZCCHC4-Flag, Flag-tagged ZCCHC4 expressing vector; ZCCHC4 Mut-Flag, Flag-tagged ZCCHC4 expressing vector with m^6^A domain catalytically inactive.


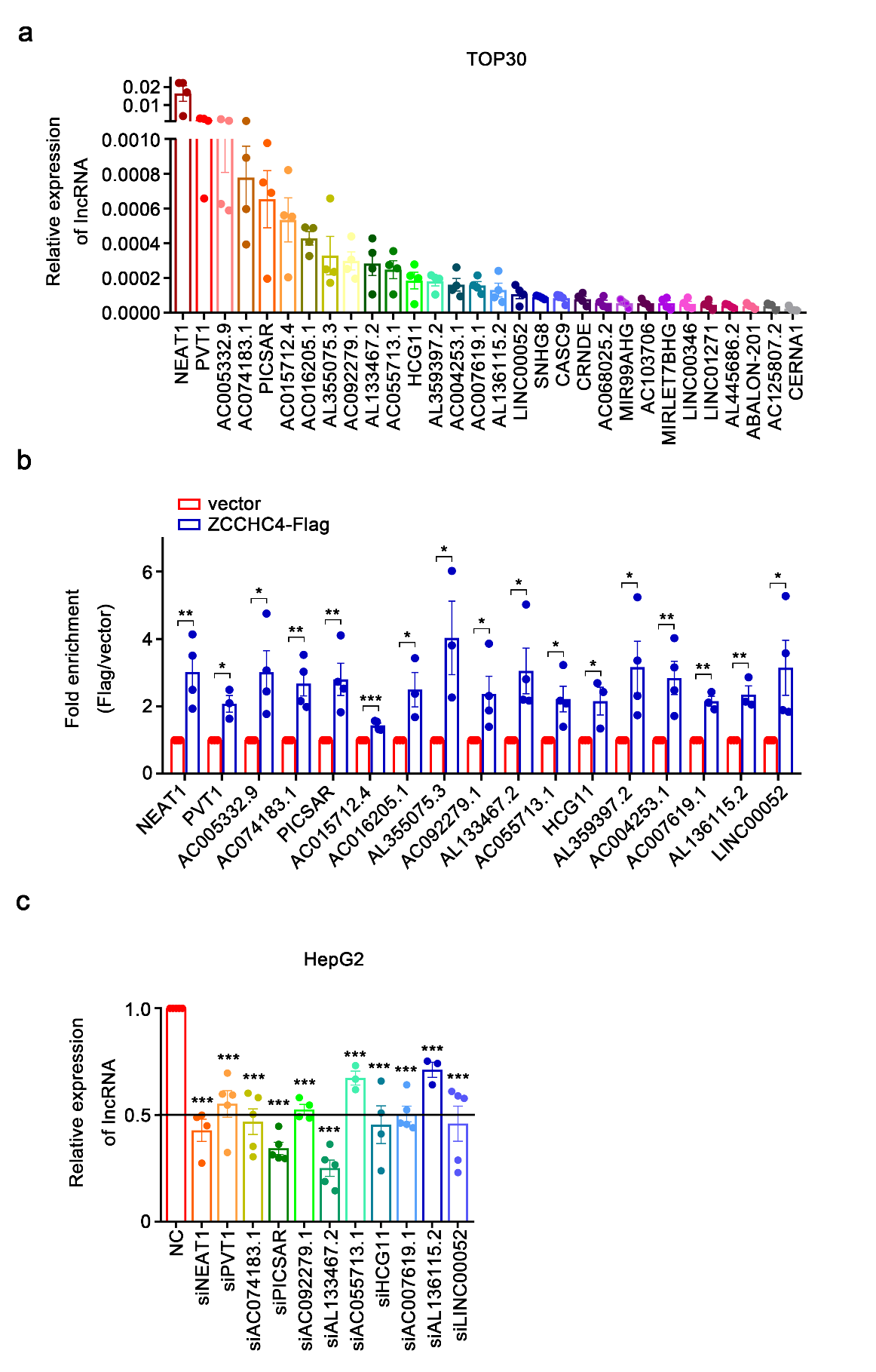


Figure. S10.

**Screen of lncRNAs interacting with ZCCHC4. a** qRT-PCR analysis of lncRNAs levels in the nucleus of HepG2 cells (n = 4 for each group). **b** RIP-qRT-PCR analysis of lncRNAs in (a) co-immunoprecipitated with anti-Flag M2 magnetic beads from ZCCHC4-Flag transfected HepG2 cells (n = 3-4 for each group). **c** qRT-PCR analysis of lncRNAs (from (b)) expression in NC- or lncRNA*-* silenced HepG2 cells (n = 3-5). Data were shown as mean ± sem (a-c). *, p < 0.05; **, p < 0.01; ***, p < 0.001 (unpaired Student’s *t* test).


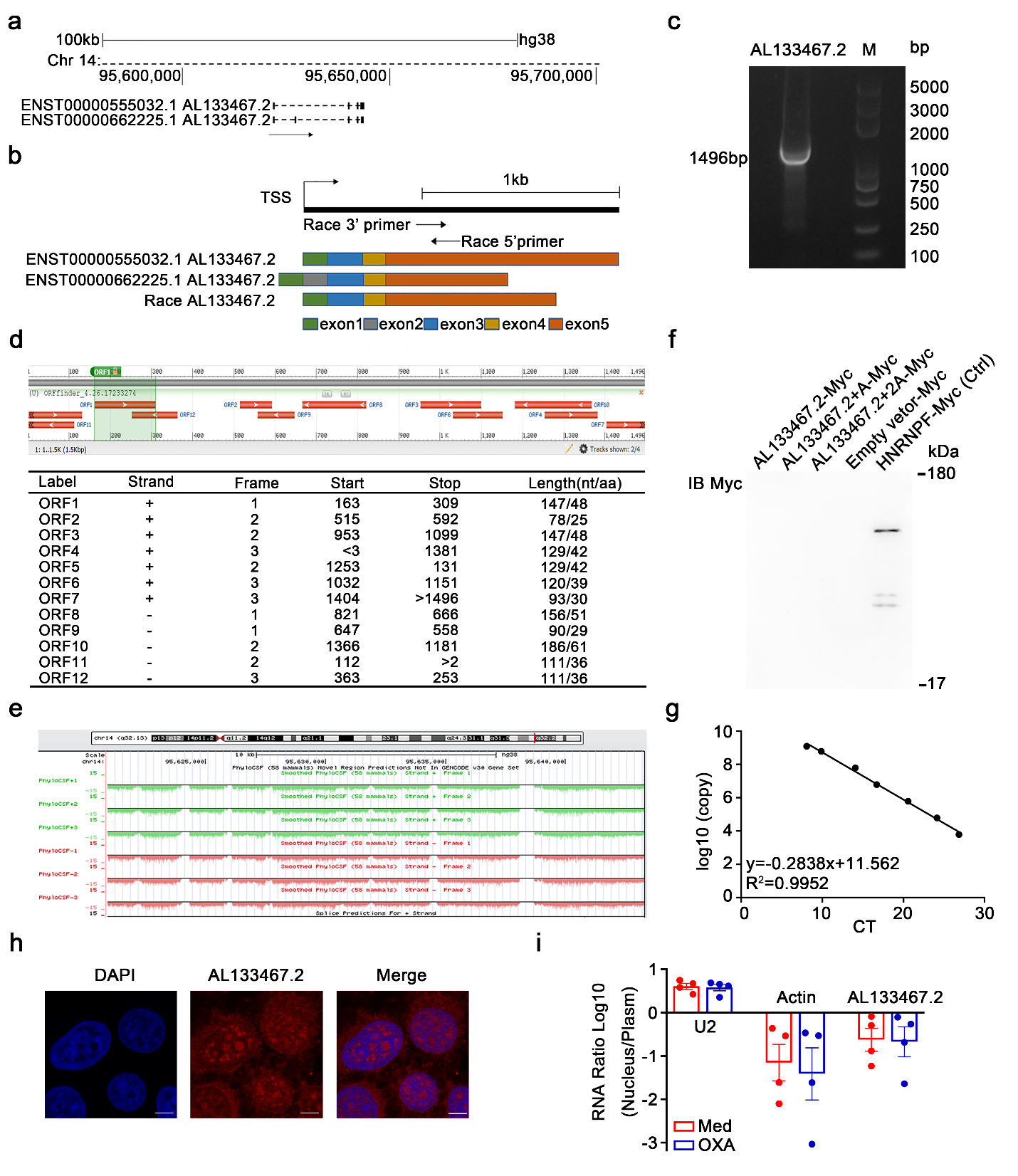


Figure. S11.

**Characterization of lncRNA AL133467.2. a** Genomic architecture of exons (filled rectangles) and introns (dotted line) of AL133467.2 based on UCSC database (http://genome.ucsc.edu/). **b, c** Primers (labeled with arrows) for RACE assay (b) and transcripts of AL133467.2 in HepG2 cells identified by RACE assay (c) were shown. **d, e** Protein coding potential prediction of AL133467.2 by using ORF Finder (d) (<http://www.ncbi.nlm.nih.gov/orffinder/>) and PhyloCSF (e). **f** Frame shift analysis for the protein coding potential of AL133467.2. One or two adenine nucleotides (a) were added to the 5’ end of AL133467.2 cDNA and cloned into the pcDNA3.1 (-B) Myc plasmid and transfected in HEK293T cells for eukaryotic expression. Immunoblot analyzing the Myc-tagged peptides expressed in cells, and Myc-tagged-HNRNPF plasmid was used as positive control. **g** Absolute copy-number analysis of AL133467.2 in HepG2 cells conducted by qRT-PCR using the standard curve. **h** Immunofluorescence images (scale bar = 5 μm) of AL133467.2 (red) in HepG2 cells. **i** qRT-PCR analysis of AL133467.2 levels in the nucleus and cytoplasm of HepG2 cells with or without OXA (62.5 µM) stimulation for 24h.


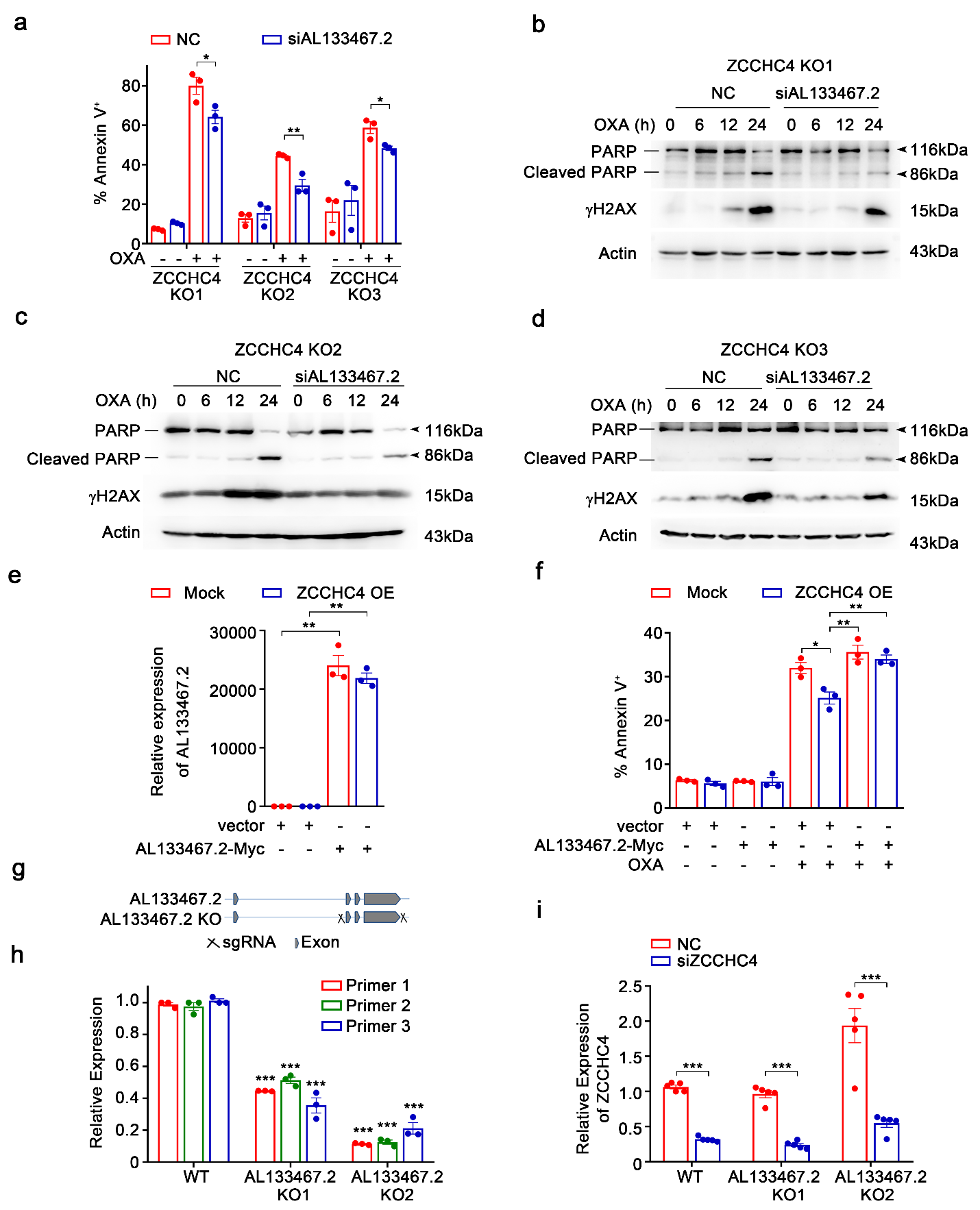


Figure. S12.

**AL133467.2 deficiency inhibits oxaliplatin-induced apoptosis in ZCCHC4 KO cells. a** Flow cytometry analyzing OXA (62.5 µM, 36 h) -induced apoptosis in NC- or AL133467.2- silenced ZCCHC4 KO cells (n = 3 for each group). NC, non-specific siRNA control. **b-d** Immunoblot analysis of cleaved PARP and γH2AX levels in NC- or AL133467.2- silenced ZCCHC4 KO cells with OXA (62.5 µM) stimulation for indicated time. **e** qRT-PCR analysis of AL133467.2 levels in empty vector or AL133467.2-Myc transfected Mock cells or ZCCHC4 OE cells (n = 3 for each group). AL133467.2-Myc, Myc-tagged AL133467.2 expressing vector; ZCCHC4 OE**,** cells stably overexpressing ZCCHC4. **f** Flow cytometry analyzing OXA (62.5 µM, 36 h) -induced apoptosis in empty vector or AL133467.2-Myc transfected Mock cells or ZCCHC4 OE cells (n = 3 for each group). **g** Strategy for AL133467.2 KO cells generation. **h** qRT-PCR analysis of AL133467.2 levels in AL133467.2 KO cells using primer 1 (targeting exon 4), primer 2 (targeting exon 2) and primer 3 (targeting exon 3) (n = 3 for each group). **i** qRT-PCR analysis of ZCCHC4 levels in NC- or *ZCCHC4*- silenced AL133467.2 KO cells (n = 5 for each group). Data were shown as mean ± sem (a, e, f, h, i). *, p < 0.05; **, p < 0.01; ***, p < 0.001 (unpaired Student’s *t* test).


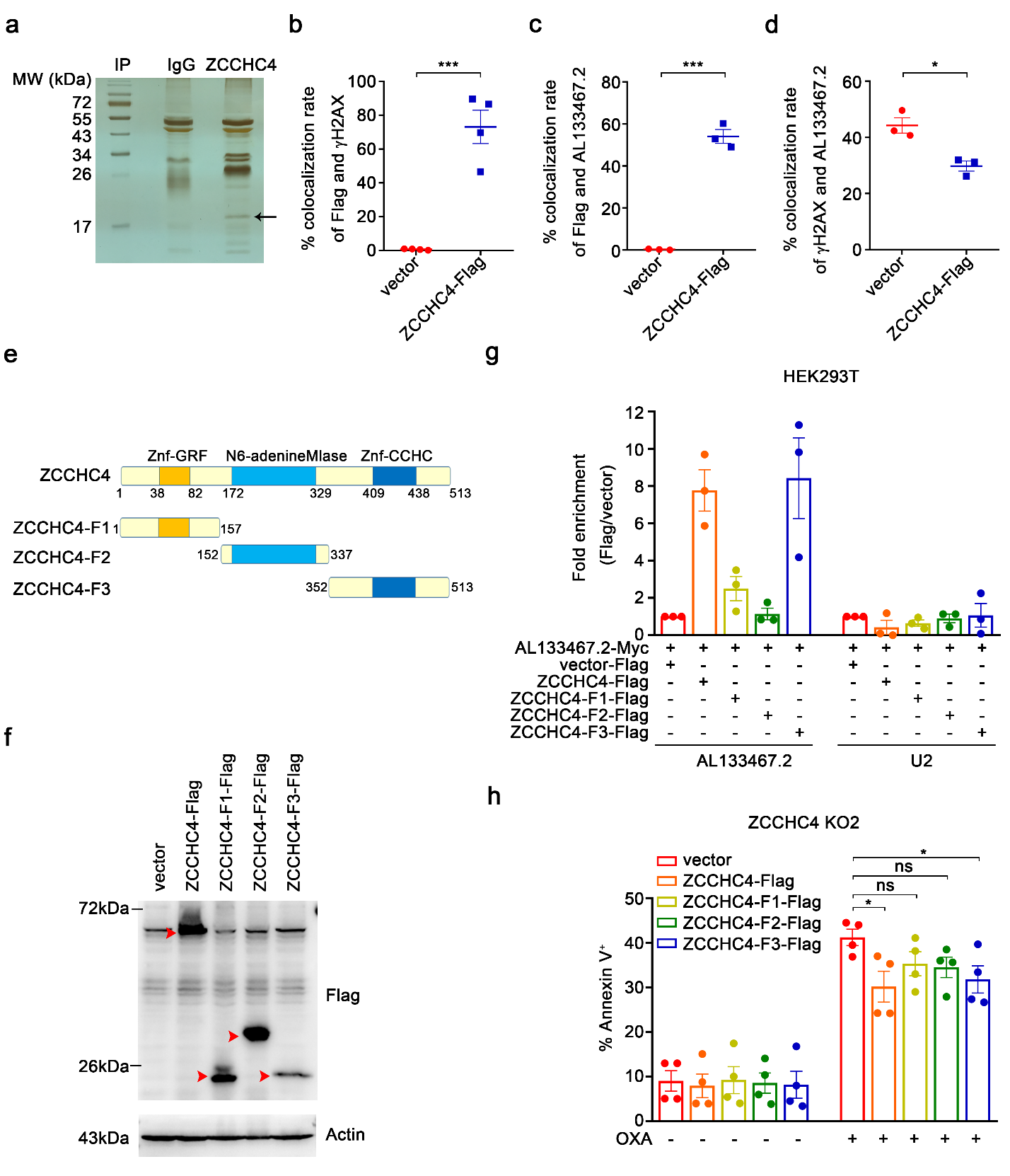


Figure. S13.

**Identification of the specific binding regions for the interaction between AL133467.2 and ZCCHC4**. **a** SDS-PAGE of immunoprecipitates from HepG2 cells with OXA (62.5 µM) treatment for 12 h using anti-IgG or anti-ZCCHC4 antibodies were stained with silver nitrate. Arrow pointed to the localization of H2AX protein. **b** Immunofluorescence assay analyzing the expression of Flag (red) and γH2AX (green) in ZCCHC4-Flag or empty vector transfected HepG2 cells with OXA (62.5 µM) treatment for 24 h, and the representative quantitative assessment of colocalization rate for the staining (n = 4 for each group) was shown. ZCCHC4-Flag, Flag-tagged ZCCHC4 expressing vector. **c, d** Immunofluorescence assay analyzing the expression of AL133467.2 (red), Flag (green) and γH2AX (magenta) in ZCCHC4-Flag or empty vector transfected HepG2 cells with OXA (62.5 µM) treatment for 12 h, and the representative quantitative assessment of colocalization rate for the staining (n = 3 for each group) was shown. **e** Schematic map for generating ZCCHC4-truncation expression plasmid. **f** Immunoblot analysis of flag-tag expression in empty vector, ZCCHC4-Flag or ZCCHC4 F1-3-Flag transfected HEK293T cells. ZCCHC4 F1-3-Flag, Flag-tagged vector with truncated fragments of ZCCHC4 expressing. **g** RIP assay was performed with anti-flag magnetic beads in HEK293T cells with co-transfected with AL133467.2-Myc and empty vector or ZCCHC4-Flag or ZCCHC4 F1-3-Flag (n = 3 per group). AL133467.2-Myc, Myc-tagged AL133467.2 expressing vector. **h** Flow cytometry analyzing OXA (62.5 µM, 36 h) -induced apoptosis in empty vector, ZCCHC4-Flag or ZCCHC4 F1-3-Flag transfected ZCCHC4 KO2 cells (n = 4 for each group). Data were shown as mean ± sem (b-d, g, h). *, p < 0.05; ***, p < 0.001; ns, not significant (b-d, h; unpaired Student’s *t* test).

Table S1.

**List of reagents**

| **Reagent** | **Source** | **Identifier** | **Usage** |
| --- | --- | --- | --- |
| **Antibodies** | | |  |
| Anti-ZCCHC4 | Abcam | Cat#: ab211325 | Immunoblot  Immunoprecipitation  Immunofluorescence |
| Anti-ZCCHC4 | Abcam | Cat#: ab154002 | Immunohistochemical |
| Anti-γH2AX | Abcam | Cat#: ab26350 | Immunoprecipitation  Immunofluorescence |
| Anti-γH2AX | Cell Signaling Technology | Cat#: 9718 | Immunoblot |
| Anti-PARP | Cell Signaling Technology | Cat#: 9532 | Immunoblot |
| Anti-cleaved caspase 3 | Cell Signaling Technology | Cat#: 9664 | Immunoblot |
| Anti-p-CHK1 | Cell Signaling Technology | Cat#: 2348 | Immunoblot |
| Anti-p-CHK2 | Cell Signaling Technology | Cat#: 2197 | Immunoblot |
| Anti-p-ATM | Cell Signaling Technology | Cat#: 5883 | Immunoblot |
| Anti- p-ATR | Cell Signaling Technology | Cat#: 2853 | Immunoblot |
| Anti-Flag | Cell Signaling Technology | Cat#: 2044 | Immunoblot  Immunofluorescence |
| Anti-p-JNK | Cell Signaling Technology | Cat#: 4668 | Immunoblot |
| Anti-JNK | Cell Signaling Technology | Cat#: 9252 | Immunoblot |
| Anti-normal rabbit IgG | Cell Signaling Technology | Cat#: 2729 | Immunoprecipitation |
| Anti-Actin | Santa Cruz | Cat#: sc-47778 | Immunoblot |
| Anti-LaminA/C | Santa Cruz | Cat#: sc-56140 | Immunoblot |
| Anti- normal mouse IgG | Santa Cruz | Cat#: sc-2025 | Immunoprecipitation |
| Anti-Ki67 | Servicebio technology | Cat#: GB13030-2 | Immunohistochemical |
| Anti-cleaved caspase 3 | Servicebio technology | Cat#: GB11532 | Immunohistochemical |
| APC Annexin V | BD Pharmingen | Cat#: 550474 | Flow cytometry |
| Anti-Flag M2 Magnetic Beads | Sigma-Aldrich | Cat#: M8823 | Immunoprecipitation |
| Anti-GAPDH | Proteintech | Cat#: HRP-60004 | Immunoblot |
| **Chemicals and assay kits** | | | |
| Oxaliplatin | Selleck | Cat#: S1224 |  |
| Doxorubicin | Sangon Biotech. | Cat#: A603456 |  |
| Protein A agrose beads | Cell Signaling Technology | Cat#: 9863 | Immunoprecipitation |
| SYTOX | ThermoFisher | Cat#: S34860 | Flow cytometry |
| Biotin RNA Labeling Mix | Roche | Cat#: 11685597910 | RNA pull-down |
| Streptavidin Agrose | ThermoFisher | Cat#: 20347 | RNA pull-down |
| T7 RNA polymerse transcription kit | Promega | Cat#: P2075 | RNA pull-down |
| Cell counting kit 8 | MCE MedChem express | Cat#: HY-K0301 | Cytotoxicity Assay |
| SMARTer RACE 5’/3’ Kit | Takara | Cat#: 634558 | RACE |
| Stellaris RNA FISH Hybridization buffer | Biosearch Technologies | Cat#: SMF-HB1-10 | FISH |
| Stellaris RNA FISH Wash Buffer A | Biosearch Technologies | Cat#: SMF-WA1-60 | FISH |
| Stellaris RNA FISH Hybridization buffer | Biosearch Technologies | Cat#: SMF-WB1-20 | FISH |
| Comet Assay Kit | Trevigen | 4250-050-k | Comet assay |

Table S2.

**List of primers sequences**

| **Name** | **Sequence (5’-3’)** |
| --- | --- |
| **sgRNA** | |
| ZCCHC4 KO sgRNA Forward | gaatgggtttgaagccgtgg |
| ZCCHC4 KO sgRNA Reverse | ccacggcttcaaacccattc |
| AL133467.2 KO1 sgRNA1 Forward | ggagaccacaagaagatggg |
| AL133467.2 KO1 sgRNA1 Reverse | cccatcttcttgtggtctcc |
| AL133467.2 KO1 sgRNA2 Forward | ggaagagattagcatttgca |
| AL133467.2 KO1 sgRNA2 Reverse | tgcaaatgctaatctcttcc |
| AL133467.2 KO2 sgRNA3 Forward | ggagaccacaagaagatggg |
| AL133467.2 KO2 sgRNA3 Reverse | cccatcttcttgtggtctcc |
| AL133467.2 KO2 sgRNA4 Forward | ccaaccctatctcaattgtc |
| AL133467.2 KO2 sgRNA4 Reverse | gacaattgagatagggttgg |
| **siRNA** | |
| ZCCHC4 Sense | GGUGACAAGAAGUCUAACATT |
| ZCCHC4 Antisense | UGUUAGACUUCUUGUCACCTT |
| NC Sense | UUCUCCGAACGUGUCACGUTT |
| NC Antisense | ACGUGACACGUUCGGAGAATT |
| **Smart silencer primers** | |
| si-h-NEAT1-1 | GCAGGTTGAAGGGAATTCT |
| si-h-NEAT1-2 | GGACCACTTAAGACGAGAT |
| si-h-NEAT1-3 | GCGAGGTGCCTTTACTACA |
| ASO-h-NEAT1-1 | GGGAGGGATGAGGGTGAAGA |
| ASO-h-NEAT1-2 | GGGACAGACAGGGAGAGATG |
| ASO-h-NEAT1-3 | AGGAGAAGGGAATGGTGGGT |
| si-h-PVT1-1 | CCCAACAGGAGGACAGCTT |
| si-h-PVT1-2 | GCTGAATGCCTCATGGATT |
| si-h-PVT1-3 | CACTGAGGCTACTGCATCT |
| ASO-h-PVT1-1 | TGGGCTTGAGCTGACCATAC |
| ASO-h-PVT1-2 | CAAGCACCTGTTACCTGTCC |
| ASO-h-PVT1-3 | AGGAGCTGCATCTACCCTGC |
| si-h-AC074183.1-1 | CGGGTGGGCAGCTTGACTT |
| si-h-AC074183.1-2 | ACCAGGCCGACCATCAACA |
| si-h-AC074183.1-3 | CGACCATCAACAGCCCTGA |
| ASO-h-AC074183.1-1 | GGTGTTGGGAAGGAGGTGAC |
| ASO-h-AC074183.1-2 | TGGGAAGGAGGTGACTTCGC |
| ASO-h-AC074183.1-3 | CCAGTGTGAGCTGTGATGGA |
| si-h-PICSAR-1 | GGACTTTCAAGAGGTAAAT |
| si-h-PICSAR-2 | GCAGTCACTTCACAGTGAA |
| si-h-PICSAR-3 | GAGCACATGTGGTGAAGAT |
| ASO-h-PICSAR-1 | GACCTCATCCATGGGAAACG |
| ASO-h-PICSAR-2 | CCAACGTGGAGCTCTAACTC |
| ASO-h-PICSAR-3 | GTCATGAGTGAAAACAGAGG |
| si-h-AC092279.1-1 | CCTAGCTTATGTGATTCTT |
| si-h-AC092279.1-2 | CCCATTACAATCTCTTCTA |
| si-h-AC092279.1-3 | ACAGAAAGTTGTGGCATAT |
| ASO-h-AC092279.1-1 | CCTGAGTATCTGGGATTACA |
| ASO-h-AC092279.1-2 | AACTCAATGCCCATCATCAT |
| ASO-h-AC092279.1-3 | GACATATTGCTGGGTTTAAC |
| si-h-AL133467.2-1 | CCAGAACAGTGTTGACTTC |
| si-h-AL133467.2-2 | AGCCTTGTGACCAGTTTCT |
| si-h-AL133467.2-3 | AGAACAGTCTAAGCAATCA |
| ASO-h-AL133467.2-1 | AAGAGATGCCTTCGTGGAC |
| ASO-h-AL133467.2-2 | AACTCCAGAACAGTGTTGAC |
| ASO-h-AL133467.2-3 | GTGATGCACACTCTTCTCTG |
| si-h-AC055713.1-1 | TAGCCAAACCAAAGATGCA |
| si-h-AC055713.1-2 | CACTTTCTCTGGGAAACTA |
| si-h-AC055713.1-3 | GGTCAGTTCTGTAATGTGA |
| ASO-h-AC055713.1-1 | ATCCATATATAAGCGGACCC |
| ASO-h-AC055713.1-2 | TCAGGGATCAGGCAGTTTAT |
| ASO-h-AC055713.1-3 | GCTCCCAAATTCTGGACCTG |
| si-h-HCG11-1 | GAATATCTGAGGTGACAAT |
| si-h-HCG11-2 | GCCTATATGTTACAAGCAT |
| si-h-HCG11-3 | GGACAAATGAGTCCTGATT |
| ASO-h-HCG11-1 | TCGTGGTGGCACATGCCTGT |
| ASO-h-HCG11-2 | CCCACTGGGTTGATGGAGAT |
| ASO-h-HCG11-3 | TTGAAGCGGTGGCGAACGTC |
| si-h-AC007619.1-1 | GAATATTTACTCTGCCCTA |
| si-h-AC007619.1-2 | CCTGACTCATCTTATTGTT |
| si-h-AC007619.1-3 | GCAACCTGGAAATCTTGAA |
| ASO-h-AC007619.1-1 | GAAGATGAAACCCTCAGCAA |
| ASO-h-AC007619.1-2 | GTGAAACTGAGATGGAACTA |
| ASO-h-AC007619.1-3 | TTGTCCAGGCTAACCTCAAA |
| si-h-AL136115.2-1 | CCTAGCTACTTACCTATAT |
| si-h-AL136115.2-2 | CCGTAGTCATTATCTTTCA |
| si-h-AL136115.2-3 | GGATGAGGTTTATACATTA |
| ASO-h-AL136115.2-1 | GCTTGAGGATAGATGAATAG |
| ASO-h-AL136115.2-2 | GCCTTTATTGGTAACTTAGG |
| ASO-h-AL136115.2-3 | ATGTTGATGTTCTCTGACTG |
| si-h-LINC00052-1 | GCAACCATCAAATCAAGAA |
| si-h-LINC00052-2 | CTGTCCTTACCTTCACTTT |
| si-h-LINC00052-3 | CCAGAGTTACAATGCAATA |
| ASO-h-LINC00052-1 | TTACTCCATATCTCTCTGTG |
| ASO-h-LINC00052-2 | CTCTGAACTATACCTCACAT |
| ASO-h-LINC00052-3 | AAACCAAATGCCAACTGGAG |
| **qRT-PCR primers** | |
| NEAT1 Forward | GATGCGCGCCTGGGTGTAGTT |
| NEAT1 Reverse | CATGCAGCCTGCCCCACTGT |
| PVT1 Forward | GCATGGAGCTTCGTTCAAGT |
| PVT1 Reverse | AGTATCCTGAAATGTGCCGGG |
| AC005332.9 Forward | CTCATGTGCTTCTTCTGGGCT |
| AC005332.9 Reverse | TTGTGTGACTGGGGCATACAG |
| AC074183.1 Forward | GTGTGAGCTGTGATGGACTTC |
| AC074183.1 Reverse | GCGGGAATACACAGCAACAC |
| PICSAR Forward | CCAAGTGTCTGCTGACTGAGA |
| PICSAR Reverse | TGCGGTCTCCTCTTGGGTA |
| AC015712.4 Forward | TTGTTTCCAGACCAGCCGAG |
| AC015712.4 Reverse | CTCGTTGTGGATGAGAGCCA |
| AC016205.1 Forward | TTTTTGGCGCGCAATGTGG |
| AC016205.1 Reverse | CAACTTGCATTGCCTCTTCTGT |
| AL355075.3 Forward | TCTCCCACAGCTCCTAGAGAA |
| AL355075.3 Reverse | GAGGGCTGAACCCCAAGTTT |
| AC092279.1 Forward | GCCTATGTTCAGCACCCGA |
| AC092279.1 Reverse | ACATGGGTACAGGATTCAGCA |
| AL133467.2 Forward | ACCTCCTGACCTGGATAGCA |
| AL133467.2 Reverse | GACAGGGACCCCACAAGTTC |
| AC055713.1 Forward | TTGACTTCCCAAGCAACCCA |
| AC055713.1 Reverse | CGCATTTTAATTCCTTGATAAGTGG |
| HCG11 Forward | GGGCTAAAGTAACACCCCTCT |
| HCG11 Reverse | CCCCACCACGCAGTGAATA |
| AL359397.2 Forward | CACAACATTGTTAAACACAACGAA |
| AL359397.2 Reverse | CCCAGCCTGGTTTGAACATT |
| AC004253.1 Forward | CTGACCAAGTCACTCAGAGCA |
| AC004253.1 Reverse | AGCTGTGGGTCTAACTGTGT |
| AC007619.1 Forward | TTCTTTTGTGGAATAAGGATTGCT |
| AC007619.1 Reverse | GGGCAGAGTAAATATTCAGTTGTGT |
| AL136115.2 Forward | TGACTGAGCCAGCTGTAAAGA |
| AL136115.2 Reverse | TTCACACACACTTCTCCCCAAA |
| LINC00052 Forward | CCAACTGGAGTTCGCCTACA |
| LINC00052 Reverse | TTAAAGAGGCGTAGGAGGGGA |
| SNHG8 Forward | CTATAAGAGAGCGTGTGCCGA |
| SNHG8 Reverse | GCCCCCGACCTACCAATATC |
| CASC9 Forward | TTGGTCAGCCACATTCATGGT |
| CASC9 Reverse | GCCAATGACTCTCCAGCCAA |
| CRNDE Forward | ACACGGCTTTCCGGAGTAGA |
| CRNDE Reverse | GCCAACATTTGGAGGAACCC |
| AC068025.2 Forward | ATCTTCAGCTTAGGTAGTGAAAAAG |
| AC068025.2 Reverse | TTTACATGCCCAGCCAACTT |
| MIR99AHG Forward | CCCTAGGCCATGAGTCAACA |
| MIR99AHG Reverse | TGACCTCAGCCTCCTCCATT |
| AC103706.1 Forward | CCTGACGCCCTTGGTTTTTG |
| AC103706.1 Reverse | AGGGGACAGTAGCCGATGTA |
| MIRLET7BHG Forward | GGCGCCTTCAGCTTGAGATA |
| MIRLET7BHG Reverse | CTTCTTGACCTGAGGGTGGAC |
| LINC00346 Forward | AGTGTCATCAGATCCACGGC |
| LINC00346 Reverse | CCAGTGTCTGTCCCTCCCTA |
| LINC01271 Forward | AGCTCGTCTCTGCACATTGTA |
| LINC01271 Reverse | TGGGATCTAGGGAGCTGACTC |
| AL445686.2 Forward | AAGAATCCTCCCACCTCATGC |
| AL445686.2 Reverse | CAAGGCAACCCAGGGACTAC |
| ABALON-201 Forward | CTTGGCTCTCCGCCTCCTAC |
| ABALON-201 Reverse | CCTGCGTCCCTCACTGAAAC |
| AC125807.2 Forward | GAGCCCTGTGGGGATAGAGA |
| AC125807.2 Reverse | GCCAATGGGAAACAGTCTGC |
| CERNA1 Forward | ACGCTCTTCGGTTTGTTTCAG |
| CERNA1 Reverse | GAAACAGCCGTCCATCCCTAA |
| U2 Forward | GGCTAAGATCAAGTGTAGTATCTGTTC |
| U2 Reverse | GCTCCTATTCCATCTCCCTGCTC |
| Actin Forward | CATGTACGTTGCTATCCAGGC |
| Actin Reverse | CTCCTTAATGTCACGCACGAT |
| ZCCHC4 Forward | CCCGTGCGTATTTTCACCAA |
| ZCCHC4 Reverse | GGTTCCATTTCCTGCCATCC |
| AL133467.2 Primer1 Forward | ACCTCCTGACCTGGATAGCA |
| AL133467.2 Primer1 Reverse | GACAGGGACCCCACAAGTTC |
| AL133467.2 Primer2 Forward | GCCTACACACAGAGCTTCCC |
| AL133467.2 Primer2 Reverse | AATCCTCCCAGCTGGTTCAC |
| AL133467.2 Primer3 Forward | TTCGTGGACGGATGAAGCTC |
| AL133467.2 Primer3 Reverse | CCAAACTGCTGCCTCATCCTA |
| **Probes for AL133467.2** | |
| Probe #1 | gagtatgatgctgctgatgg |
| Probe #2 | atctcttacacccacaagat |
| Probe #3 | ccttaactacgagctggaga |
| Probe #4 | ctaggtccccagaattaatt |
| Probe #5 | agagaagagtgtgcatcacc |
| Probe #6 | ttagactgttctgctagtgt |
| Probe #7 | gacaactctctgtccttcag |
| **RACE primers for AL133467.2** | |
| 5’RACE primer | GATTACGCCAAGCTTggcatcatgctacatgctggtgtgaccc |
| 3’RACE primer | GATTACGCCAAGCTTacaggccagccccacagaagagaaggcct |

References

1. Hou, J. *et al*. Hepatic RIG-I predicts survival and interferon-α therapeutic response in hepatocellular carcinoma*. Cancer Cell*. **25**, 49-63 (2014).

2. Tang, Z. *et al*. GEPIA: a web server for cancer and normal gene expression profiling and interactive analyses*.* *Nucleic Acids Res*. **45**, W98-W102 (2017).

3. Subramanian, A. *et al*. Gene set enrichment analysis: a knowledge-based approach for interpreting genome-wide expression profiles*.* *Proc Natl Acad Sci U S A*. **102**, 15545-50 (2005).

4. Chen, W. *et al*. Induction of Siglec-G by RNA viruses inhibits the innate immune response by promoting RIG-I degradation*.* *Cell*. **152**, 467-78 (2013).

5. Liu, J. *et al*. CCR7 Chemokine Receptor-Inducible lnc-Dpf3 Restrains Dendritic Cell Migration by Inhibiting HIF-1alpha-Mediated Glycolysis*.* *Immunity*. **50**, 600-615 (2019).
